# Associations Between Digital Health Intervention Engagement, Physical Activity, and Sedentary Behavior: Systematic Review and Meta-analysis

**DOI:** 10.2196/23180

**Published:** 2021-02-19

**Authors:** Matthew Mclaughlin, Tessa Delaney, Alix Hall, Judith Byaruhanga, Paul Mackie, Alice Grady, Kathryn Reilly, Elizabeth Campbell, Rachel Sutherland, John Wiggers, Luke Wolfenden

**Affiliations:** 1 School of Medicine and Public Health University of Newcastle Callaghan Australia; 2 Hunter New England Population Health Wallsend Australia; 3 Hunter Medical Research Institute New Lambton Heights Australia; 4 Priority Research Centre for Heath Behaviour University of Newcastle Callaghan Australia; 5 School of Health Sciences and Priority Research Centre for Stroke and Brain Injury, University of Newcastle Callaghan Australia; 6 Centre for Research Excellence in Stroke Recovery and Rehabilitation, Florey Institute of Neuroscience Melbourne Australia

**Keywords:** engagement, adherence, digital health intervention, digital behavior change intervention, physical activity, sedentary behavior, mobile phone

## Abstract

**Background:**

The effectiveness of digital health interventions is commonly assumed to be related to the level of user engagement with the digital health intervention, including measures of both digital health intervention use and users’ subjective experience. However, little is known about the relationships between the measures of digital health intervention engagement and physical activity or sedentary behavior.

**Objective:**

This study aims to describe the direction and strength of the association between engagement with digital health interventions and physical activity or sedentary behavior in adults and explore whether the direction of association of digital health intervention engagement with physical activity or sedentary behavior varies with the type of engagement with the digital health intervention (ie, subjective experience, activities completed, time, and logins).

**Methods:**

Four databases were searched from inception to December 2019. Grey literature and reference lists of key systematic reviews and journals were also searched. Studies were eligible for inclusion if they examined a quantitative association between a measure of engagement with a digital health intervention targeting physical activity and a measure of physical activity or sedentary behavior in adults (aged ≥18 years). Studies that purposely sampled or recruited individuals on the basis of pre-existing health-related conditions were excluded. In addition, studies were excluded if the individual engaging with the digital health intervention was not the target of the physical activity intervention, the study had a non–digital health intervention component, or the digital health interventions targeted multiple health behaviors. A random effects meta-analysis and direction of association vote counting (for studies not included in meta-analysis) were used to address objective 1. Objective 2 used vote counting on the direction of the association.

**Results:**

Overall, 10,653 unique citations were identified and 375 full texts were reviewed. Of these, 19 studies (26 associations) were included in the review, with no studies reporting a measure of sedentary behavior. A meta-analysis of 11 studies indicated a small statistically significant positive association between digital health engagement (based on all usage measures) and physical activity (0.08, 95% CI 0.01-0.14, SD 0.11). Heterogeneity was high, with 77% of the variation in the point estimates explained by the between-study heterogeneity. Vote counting indicated that the relationship between physical activity and digital health intervention engagement was consistently positive for three measures: subjective experience measures (2 of 3 associations), activities completed (5 of 8 associations), and logins (6 of 10 associations). However, the direction of associations between physical activity and time-based measures of usage (time spent using the intervention) were mixed (2 of 5 associations supported the hypothesis, 2 were inconclusive, and 1 rejected the hypothesis).

**Conclusions:**

The findings indicate a weak but consistent positive association between engagement with a physical activity digital health intervention and physical activity outcomes. No studies have targeted sedentary behavior outcomes. The findings were consistent across most constructs of engagement; however, the associations were weak.

## Introduction

Physical activity of any intensity reduces the risk of death and noncommunicable diseases [1]. Sedentary behavior is highly prevalent, displaces time to be physically active, and is associated with noncommunicable diseases and premature death [2,3]. As such, efforts to increase physical activity and concurrently decrease sedentary behavior have been identified internationally as public health priorities [4].

Digital health interventions (DHIs) have the potential to address physical inactivity, as they are accessible by large proportions of the population and can be delivered with high effectiveness at a low cost [5,6]. The World Health Organization defines *digital health* as the use of digital, mobile, and wireless technologies to support the achievement of health objectives and is inclusive of both mobile health (mHealth) and eHealth [7], including mobile phones, portable computer tablets (eg, iPads), web-based interventions, smartphone apps, and wearable devices [8]. An attractive feature of DHIs is their capacity to be scaled for large populations while concurrently being tailored to specific target groups [7,9]. There has been a considerable investment in the development and research on DHIs to improve physical activity, with a rise of 26% per year in journal article publications since 2000 [10]. Furthermore, the use of DHIs to promote and support participation in physical activity has been recommended in the Global Action Plan on Physical Activity 2018-2030 [4,11].

Despite the significant growth in DHIs, there is a limited understanding of the extent to which DHIs impact physical activity outcomes. Overall, systematic reviews indicate that DHIs targeting adult populations may have a modest effect in improving physical activity when delivered web based [12-16] and through smartphone applications [12,13,17-19]. A recent meta-analysis found that per week, web-based interventions increased moderate-to-vigorous intensity physical activity by 13.4 minutes (95% CI 12.96-13.89) and steps by 2185 (95% CI 1765-2605) [15]. Similarly, another meta-analysis of web-based interventions targeting physical activity found that physical activity significantly improved in the short term (Cohen *d*=0.14). The review suggested that the small effect size may be attributed to a lack of engagement with web-based interventions [14]. Other meta-analyses of mobile phone app interventions have reported effects that favored the intervention but were not statistically significant [17,19], with the suggestion that a lack of engagement may explain the lack of evidence to support effectiveness.

Participant exposure needs to be sufficient for any DHI to have an effect [20]. Engagement has been defined as the (1) extent of DHI *usage* such as the frequency, duration, amount, and depth of the accessed DHI and (2) *subjective experience* characterized by attention, interest, and affect [20]. To our knowledge, only one systematic review has explored the association between objective levels of engagement with DHIs (usage measures) and physical activity or sedentary behavior [8]. The review by Donkin et al [8] explored the association between the level of engagement with DHIs (web-based interventions) targeting adults and a range of health outcomes [8]. Included studies predominately reported the measures of psychological health, dietary intake, weight management, and smoking. The results were reported narratively because of the diverse measures of engagement and health outcomes. Only 3 of the 33 studies included measures of physical activity (n=5 associations) [21-24], with no studies reporting sedentary behavior outcomes. The engagement measures explored by the 3 studies included in the Donkin review focused on logins (n=3), activities completed (n=1), and website exposure (n=1). Of the 3 physical activity studies, Marcus et al [22] showed that a higher number of logins were correlated with an increase in the physical activity from baseline to 12 months. Similarly, McKay et al [21] found that those who logged into the program on 3 or more occasions had greater increases in physical activity than those with fewer logins. McKay et al [21] additionally found website exposure (usage) to be associated with higher increases in physical activity. In contrast, Carr et al [23,24] showed that neither the number of logins nor the number of activities completed were associated with physical activity at 8 months.

Understanding the relationship between engagement and health outcomes is important because it provides an opportunity to optimize the impact of interventions [20,25]. Engagement is hypothesized to influence the relationship between a DHI and the mechanisms of action of the DHI (eg, skills, attitudes, beliefs, knowledge), which then leads to the target behavior (eg, physical activity) [20]. Even smaller improvements in the effectiveness of DHIs are important, given the potential reach of these interventions [6,10]. The previous review by Donkin et al [8] used a definition of engagement that focused on the usage and user-directed web-based interventions, which excluded smartphone apps and group-based DHIs [8]. Since 2010, when their search was conducted, there has been a large increase in the mobile- and app-based research applied to physical activity; hence, there is an increased opportunity to garner further understanding of this relationship [8,10]. A broader definition of engagement that encompasses subjective experience has also been developed [20]. To our knowledge, no review has explored the relationship between subjective experience with DHIs and physical activity or sedentary behavior [20]. Therefore, a more contemporary review of the evidence is warranted.

### Objective

In this context, we aim to (1) describe the direction and strength of the association between engagement with DHIs and physical activity and/or sedentary behavior in adults and (2) explore whether the direction of association between DHI engagement and physical activity or sedentary behavior varies by the type of engagement (ie, subjective experience, activities completed, time, and logins).

## Methods

### Design

This review was prospectively registered with the International Prospective Register for Systematic Reviews (PROSPERO; CRD42018110657) and is reported in accordance with the Joanna Briggs Institute guidance for conducting systematic reviews of association [26].

### Search Strategy

Searches for peer-reviewed literature were undertaken with the assistance of a research librarian in 4 electronic databases: Embase, MEDLINE, PsycINFO, and Scopus (Multimedia Appendix 1). We searched for records from the database inception to December 2019. Searches were restricted to English. This review was conducted alongside another review that aimed to describe the association between DHI engagement and dietary intake (PROSPERO CRD42018112189 [27]). Therefore, *dietary intake* search terms were also included in the search strategy. We used the modified versions of published search filters for physical activity and sedentary behavior [28], engagement [20], and DHIs [20,29,30].

#### Additional Search Methods

We conducted hand searches of all the publications from January 2016 to December 2019 in the following journals: *Journal of Medical Internet Research*, *JMIR mHealth and uHealth*, *JMIR Medical Informatics*, and *JMIR Public Health and Surveillance*. We conducted gray literature searches in “Google.com/ncr” search engine and used the search terms *Physical Activity* or *Sedentary Behavior* and *Engagement* and *Digital Health Intervention* and screened the first 200 hits for relevance. We screened the reference lists of key systematic reviews of DHI engagement [8,20]. We also contacted the authors of included studies for other potentially relevant studies.

### Inclusion Criteria

#### Types of Studies

We included study designs that examined a quantitative association between a measure of engagement with a DHI and a measure of physical activity and/or sedentary behavior. DHIs were defined as the use of digital, mobile, and wireless technologies to support the achievement of health objectives [7], inclusive of both mHealth and eHealth. We adopted Perski et al’s [20] definition of engagement, defined as both the extent of the usage of the DHI (amount, frequency, duration, and depth; eg, activities completed, time, and logins) and the subjective experience (characterized by attention, interest, and affect). DHIs included but were not limited to mobile phones, portable computer tablets (eg, iPads), web-based interventions, and smartphone apps. We included DHIs involving synchronous communication as part of the program (eg, web-based chat, teleconferencing). We applied no restrictions on the length of the follow-up period or the country of origin of the studies. We included studies that recruited participants in the real-world settings (ie, ecological studies) as well as nonecological studies (ie, those conducted under controlled research conditions, where repeated contacts with research staff, comprehensive assessments, and recruitment to the study occurs before the individual accessing the DHI) [31]. All the quantitative study designs were also included.

#### Population

We included any study undertaken with adult users (aged ≥18 years) of a DHI targeting physical activity or sedentary behavior. The studies of participants who had access to a DHI and the opportunity to engage with the DHI were eligible.

#### Exposure

We included studies reporting any measure of engagement with a DHI, defined as the extent of usage (eg, activities completed, time, and logins) or the subjective experience of users (eg, measures of attention, interest, and affect, including but not limited to enjoyment, satisfaction, user experience, and usability) [20]. Engagement can be collected by the DHI (eg, analytics), observation, surveys of DHI users, or other quantitative methods. We excluded the qualitative measures of engagement (eg, focus groups).

#### Outcome

We included studies reporting any measure of physical activity or sedentary behavior, including but not limited to self-report (eg, minutes of moderate-to-vigorous physical activity, minutes of walking, self-reported steps, distance traveled) and measured by a device (eg, steps from pedometer, mobile phone data, accelerometers). These could be reported in specific settings (eg, while at work), periods of the day (eg, mornings), or as the whole day. We included both cross-sectional measures of physical activity (ie, one time point) and those studies with multiple time points calculating changes in the physical activity over time (ie, cohort studies).

### Exclusion Criteria

We excluded the following studies:

- Case studies, letters to the editor, and qualitative studies.
- Studies that targeted children and adolescents (<18 years).
- Studies that purposely sampled or recruited individuals based on pre-existing health-related conditions, including chronic health conditions such as chronic pain, a diagnosis of chronic disease, communicable disease, or mental illness, given our interest in generalizing the findings to general community samples.
- Studies in which the individuals engaging in the DHI were not the target of the physical activity or sedentary behavior intervention (eg, doctors engaging with a physical activity app for their patients).
- Studies that included a non-DHI component within an intervention (eg, a face-to-face component and digital components). This step was taken to ensure that the measures of engagement reflected only the digital component and not the intervention more generally.
- Interventions not functioning at computer- or internet-based capacity (eg, SMS, CD-Rom, and computer-based interventions) to focus on more contemporary DHIs.
- Those that targeted multiple health behaviors for the prevention of chronic disease (eg, sleep and physical activity or diet and physical activity) to reduce heterogeneity between health behaviors.
- Studies where the full text was not available.

### Data Collection and Analyses

#### Selection of Studies

**Figure 1:**
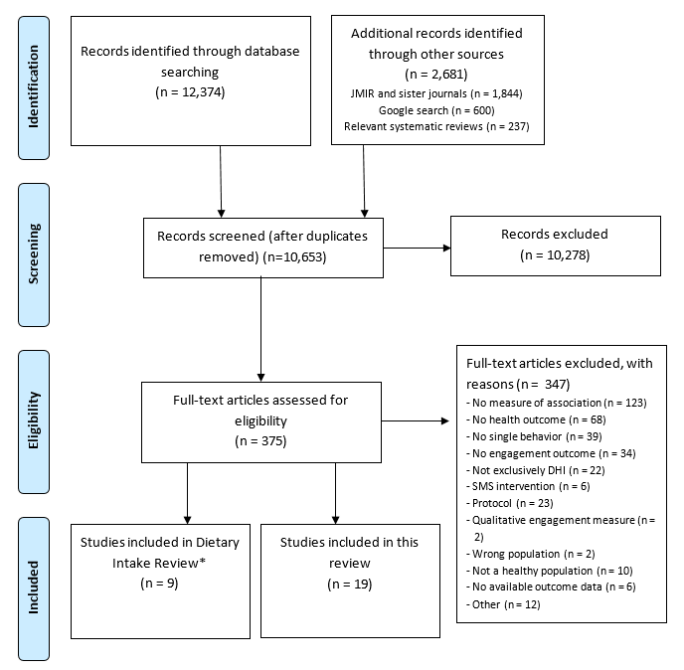
PRISMA (Preferred Reporting Items for Systematic Reviews and Meta-Analyses) flow diagram. *This review was conducted alongside another review aiming to describe the association between DHI engagement and dietary intake (PROSPERO CRD42018112189). Therefore, ‘dietary intake’ search terms were also included in the search strategy, but removed for reporting elsewhere.

After removing the duplicates, the authors (MM, TD, and JB) single-screened titles and abstracts for potentially eligible studies using Covidence. At title and abstract screening, we included studies in the full text review when the abstract reported both a physical activity and/or sedentary behavior outcome as well as a DHI engagement outcome (including meeting other inclusion and exclusion criteria). Therefore, studies that did not report a measure of association between physical activity and/or sedentary behavior and DHI engagement were still included for a full text review. This was done to ensure that the studies were not excluded in error. This screening process was implemented after an initial pilot screening of 100 full texts by MM, who found that none of the abstracts that reported only a health outcome or only an engagement outcome were incorrectly screened out. Following the title and abstract review, we obtained full texts of all potentially relevant or unclear articles, and authors (MM, TD, AG, and KR) independently reviewed these against our inclusion criteria. Reasons for exclusion were recorded in a *characteristics of excluded studies* table. The review authors were not blinded to author or journal information. The number of articles identified, screened, eligible, and included were recorded according to the PRISMA (Preferred Reporting Items for Systematic Reviews and Meta-Analyses) statement [32] (Figure 1).

#### Data Extraction and Management

Pairs of review authors (MM and TD) independently extracted data using a data extraction form adapted from the Cochrane Public Health Group Methods Manual and used previously by the research team [33]. Given the complexity of the review, all the extracted data were reviewed by an experienced statistician and methods expert, and any disagreements were resolved by the third author (AH). In case of missing study data, we attempted to contact the corresponding authors to obtain the required information. The following data were extracted:

- Study characteristics including authors’ name, year of publication, overall study design, intervention target (ie, intended intervention recipient), recruitment method (ecological or nonecological), and sample size.
- Characteristics of the intervention, including type of DHI (ie, web-based, smartphone app, Exergame, and Facebook group), description of DHI components, and length of exposure to DHI.
- Outcomes including both a description of the physical activity or sedentary behavior measure and the engagement measure; the analysis method used to examine associations including adjustments for confounding (for quality assessment); magnitude of the association (ie, odds ratio [OR] or regression coefficient or estimate along with a measure of variability [if available], 95% CIs or standard deviation or standard error); statistical significance of the association; and further information to allow quality assessment.

#### Critical Appraisal

Pairs of review authors (MM, PM, and RS) assessed the methodological quality of studies independently using the Newcastle Ottawa Scale for cohort studies [34] (n=13 studies) and cross-sectional studies [35] (n=6 studies). We defined cross-sectional studies as the ones using a single time point of data for measuring the physical activity or sedentary behavior (eg, follow-up), whereas cohort studies as those that used multiple time points of data and calculated changes over time (eg, change from baseline to follow-up). All the studies were assessed based on their highest quality measure of association (ie, final follow-up time point, adjusted, device-measured physical activity or sedentary behavior, and objectively assessed engagement were preferred to mid-point follow-up, unadjusted, self-reported physical activity or sedentary behavior, and self-reported engagement).

The Newcastle Ottawa Scales utilizes a star system to assess the methodological quality of studies. The cohort tool assigns a maximum of 9 points for quality assessment in 3 domains: (1) selection of study groups (up to 4 points), (2) comparability of these groups (up to 2 points), and (3) assessment of outcomes (up to 3 points). The cross-sectional tool assigns a maximum of 10 points across the same 3 domains: (1) selection of study groups (up to 5 points), (2) comparability of these groups (up to 2 points), and (3) assessment of outcomes (up to 3 points; Multimedia Appendices 2 and 3 for scoring systems, adopted from Wells et al [34] and Modesti et al [35]).

Within the cohort tool, for the item “was follow up long enough for outcomes to occur,” studies were awarded a point if they had a minimum follow-up period of 3 months. This period was chosen based on the current evidence on the length of DHI engagement, where those that are designed to be used for 3 months or longer tend to be more effective than those designed for shorter durations [14,15]. Within the cross-sectional tool, for the item “the study controls for the most important factor (select one),” we selected age as the factor to control for, as it is unanimously possible to control for this across studies and is an important contextual factor influencing engagement [20]. Disagreements between assessments were resolved by discussion between the pairs of review authors (MM, RS, and PM) and, where required by consulting the third review author.

#### Data Synthesis and Analysis

Data were synthesized according to the review objectives.

##### Objective 1

Describe the direction and strength of the association between engagement with DHIs and physical activity and/or sedentary behavior in adults.

We planned two separate meta-analyses: first for subjective experience and second for usage (including activities completed, time, and logins). However, we did not conduct a meta-analysis for subjective experience because of the considerable methodological heterogeneity between studies and the small number of studies reporting this outcome (n=3) [36]. Therefore, we focused on the direction and strength of the association between usage and physical activity or sedentary behavior.

A variety of different methods of association were used across the included studies. Consequently, to allow for meta-analysis, we were required to transform a number of estimates into one consistent effect index. A standardized regression coefficient was chosen as the effect index, which is a previously proposed method [37]. A list of the main transformations used for this analysis are detailed in Multimedia Appendix 4 and are predominantly based on the research works of Borenstein et al [36] and Nieminen et al [37].

We used the Dersimonian and Laird random effects method of meta-analysis to calculate a pooled standardized effect assessing the strength and direction of associations. Statistical analyses were performed using R [38]. Many studies have reported more than one association. For meta-analyses, we used the following hierarchical selection criteria to select a single association from each study for inclusion in the pooled synthesis:

- Use measures were given preference in the following order:
  activities completed, time on site then logins. This attribute reflects the level of participant involvement required for each measure of engagement, with a greater level of engagement given priority.
- Measures of total physical activity were preferred to specific physical activity or sedentary behavior types (eg, measures of physical activity of moderate-to-vigorous intensity were preferred to distance walked).
- Device-measured physical activity was preferred over self-report.
- Whole-day measures of physical activity or sedentary behavior were preferred over specific time segments (eg, whole-day physical activity was preferred to workday physical activity).
- Global scores of subjective experience engagement were preferred over the individual constructs of subjective experience (eg, engagement questionnaire score preferred to game-flow score only).
- For DHIs with team or group engagement opportunities (eg, group challenges in a step-counting website), individual measures of engagement (eg, individual logins to the DHI) were preferred to group opportunities to engage (eg, total logins from a team of DHI users), as this more accurately reflects an individual’s DHI engagement.
- Associations derived from fully adjusted models were preferred over unadjusted or partly adjusted models.

When studies did not provide sufficient data required for meta-analysis (ie, information to calculate an effect estimate and measure of variability of the effect estimate), the corresponding authors were contacted via email on up to 3 occasions and asked to provide information. When data were not available or not provided, we excluded the study from the meta-analysis. To provide supplementary data for this objective, we used vote-counting synthesis methods to describe the direction of association across these studies. We used the direction of association rather than statistical significance in accordance with the recent SWIM (Synthesis Without Meta-Analysis) guidelines [39]. We focused on a single measure of association from each study, which was selected based on the same hierarchical criteria for the selection of an association as for the meta-analysis. For vote counting, each study was summarized as either “+,” “−“ or “0.” “+” was assigned to the studies in which the point estimate and CI supported the hypothesis that higher engagement is associated with a higher physical activity or reduced sedentary behavior. “0” was assigned to the studies in which the point estimate and CI had inconclusive findings. “−“ was assigned to the studies in which the association point estimate and CI rejected the hypothesis. Several studies did not report any results, including an estimate or CI, but rather just stated whether the association was statistically significant in the hypothesized direction. In these instances, to ensure that such studies were included, we assigned either “+” or “−“ to the studies that reported *significant* association findings depending on the stated direction of the association (if provided) or “0” to the studies that reported *nonsignificant findings*.

##### Objective 2

To explore whether the direction of association between engagement and physical activity or sedentary behavior varies according to the type of engagement (ie, subjective experience, activities completed, time, and logins).

To explore whether the direction or strength of associations varied between the different types of engagement, we classified each association across all the studies as either subjective experience, activities completed, time (time spent using the intervention; eg, session duration), or logins. Studies could contribute more than one association but only one association for each of the four types of engagement.

When studies had more than one association for a given type of engagement, we gave preference to the measures of association from adjusted associations and excluded the measures of association from the self-report measures of physical activity or sedentary behavior where device measures were available for the same engagement variable. Finally, for studies reporting associations at multiple time points, we included only data from the final time point.

We used vote-counting methods to explore the direction of the association between each type of engagement and physical activity or sedentary behavior outcomes. Each association was summarized as either “+,” “−,” or “0,” following the same procedures described above, with “0” being assigned to studies with associations reporting mixed findings.

## Results

### Search Results

The searches resulted in 13,192 potentially relevant abstracts. After removing the duplicates, 10,653 unique citations were retained for review. After the title and abstract screening, 375 full texts were identified and screened. Overall, 19 studies were included in our review (Figure 1).

### Study Characteristics

Detailed characteristics of each study (n=19) [22,24,40-56], and each measure of association, are provided in Multimedia Appendix 5. All 19 studies (n=7776 participants) were of physical activity measures, with no study using measures of sedentary behavior. Of the 19 studies, 12 were web-based interventions [22,24,41,44,45,47-50,52,54,55], 5 were app-based [43,46,51,53,56], and the remaining were Facebook-based (n=1) [42] and exergames (n=1) [40]. Cohort designs were used in 13 studies [22,24,42-44,47,48,50-52,54-56]. The remaining 6 studies used cross-sectional designs [40,41,45,46,49,53]. Across both cohort and cross-sectional studies, 11 studies included an analysis of the intervention arm of a randomized controlled trial [22,24,42-45,48,50,52,54,55].

The majority of studies included the usage measures of engagement (ie, activities completed, time, or logins; n=18), whereas 3 studies included subjective experience measures of engagement [40,46,48]. Participants across all the studies were predominately female (71%). Most studies used nonecological recruitment methods (n=11), with the remaining 8 studies using a mixture of ecological and nonecological recruitment methods [17,44,46,49,51,53,55,56]. The sample sizes across all the studies ranged from 7 to 3555 (mean 389; SD 760.6).

### Critical Appraisal (Quality Assessment)

Of the 19 studies, almost half were assessed to be of *poor* quality (n=9). Two studies were considered to be of fair quality, and the remaining studies (n=8) were considered to be of good quality. Quality assessment results for cohort studies are summarized in Table 1, and quality assessment results for cross-sectional studies are summarized in Table 2.

**Table 1:** Quality assessment (Newcastle-Ottawa Quality Assessment Scale criteria for cohort studies).

Study	Selection				Comparability	Outcome			Selection^a^	
	Representativeness of the exposed cohort	Selection of the nonexposed cohort	Ascertainment of exposure	Outcome not present at the start of study	Cohort statistical analysis	Assessment of the outcome	Was follow-up long enough for outcomes to occur	Adequacy of follow-up cohorts		
Carr et al [24]	★	★	★	★	0	0	★	0		Poor
Edney et al [42]	★	★	★	0	0	0	0	★		Poor
Edney et al [43]	★	★	★	★	★	★	★	★		Good
Ferney et al [44]	★	★	★	0	★	0	★	★		Good
Kwan et al [47]	★	★	★	0	0	0	0	★		Poor
Lewis et al [48]	★	★	★	★	★	0	★	★		Good
Linke et al [50]	★	★	★	★	★	★	★	★		Good
Ma et al [51]	★	★	★	0	★	★	0	★		Good
Maher et al [52]	★	★	★	0	★	0	0	★		Poor
Marcus et al [22]	★	★	★	★	★	0	★	★		Good
Rebar et al [54]	★	★	★	0	★★	0	★	0		Poor
Wanner et al [55]	★	★	★	0	★	0	★	0		Poor
Xian et al [56]	★	★	★	★	★	★	0	★		Good

^a^Quality score: Overall scores were given (good, fair, and poor). Good quality: 3 or 4 stars (★) in the selection domain AND 1 or 2 stars in the comparability domain and 2 or 3 stars in the outcome domain; Fair quality: 2 stars in the selection domain and 1 or 2 stars in the comparability domain and 2 or 3 stars in the outcome/exposure domain; poor quality: 0 or 1 star in the selection domain OR 0 stars in the comparability domain OR 0 or 1 stars in the outcome/exposure domain.

**Table 2:** Quality assessment (Newcastle-Ottawa Quality Assessment Scale criteria for cross-sectional studies).

Study	Selection				Comparability	Outcome		Quality score^a^
	Representativeness of the exposed cohort	Sample size	Comparability of nonrespondents	Ascertainment of the exposure	Statistical analysis design features	Assessment of outcome	Statistical test	
Bronner et al [40]	0	★	0	★	0	★★	0	Poor
Davies et al [41]	★	0	★	★	★★	★	0	Poor
Hansen et al [45]	★	★	★	★	0	★	0	Poor
Hoj et al [46]	★	0	0	★	★	★	★	Fair
Lieber et al [49]	★	0	★	★	★	★	★	Good
Marquet et al [53]	★	0	0	★	★	★★	★	Fair

^a^Quality score: Overall scores were given (good, fair, and poor). Good quality: 3 or 4 stars (★) in the selection domain AND 1 or 2 stars in the comparability domain and 2 or 3 stars in the outcome domain; fair quality: 2 stars in the selection domain and 1 or 2 stars in the comparability domain and 2 or 3 stars in the outcome/exposure domain; poor quality: 0 or 1 star in the selection domain OR 0 stars in the comparability domain OR 0 or 1 stars in the outcome/exposure domain.

#### Selection

Within the cohort studies (n=13), all studies scored highly in the selection domain. Representativeness of the sample was high, with all studies scoring a star for being either truly or somewhat representative of the average target population. The nonexposed cohort was drawn from the same community as the exposed cohort in all studies. The exposure (engagement) was usually measured using either objective measurement (eg, Google Analytics) or self-report.

Within cross-sectional studies (n=6), all but one study had somewhat representative or truly representative samples. Sample size calculations were often not provided (n=4). Nonresponse characteristics were not provided or poorly described in half of the studies. No studies used validated measurement tools; however, the tool was made available or well described in all studies.

#### Comparability

Cohort studies controlled for confounders in 10 of the 13 studies. However, only one study controlled for all 3 factors (ie, age, sex, and marital status) required to score two stars. Therefore, most studies scored one star. Three studies scored zero stars, as they used unadjusted analyses.

In the cross-sectional studies, only 2 studies used adjusted analyses. One further study controlled for age and scored an additional star.

#### Outcome

Within the cohort studies, four studies used an independent blind assessment or record linkage (eg, steps via a mobile phone). The remaining studies scored zero stars as they used self-reporting. Eight studies were followed up after a sufficient duration (3 months); therefore, they scored a star. Five studies had follow-up shorter than 3 months. The follow-up cohort rate was inadequate in 3 studies, as no description of differences in responders and nonresponders was provided, and less than 80% responded. The remaining 10 studies scored a star, as either more than 80% responded at follow-up, or there were no differences in responders and nonresponders.

Of the cross-sectional studies, most studies used self-reported physical activity measurements (n=4). Half of the studies were considered to have used appropriate and well-described statistical tests; the remaining studies did not describe or provide sufficient details (eg, measures of variance).

### Objective 1

Describe the direction and strength of the association between engagement with DHIs and physical activity or sedentary behavior in adults.

Although we had planned two meta-analyses, one for each of the conceptually different forms of engagement (use and subjective experience) [20], we did not conduct a meta-analysis for subjective experience because of the considerable methodological heterogeneity among the studies and the small number of studies reporting this outcome (n=3) [36]. Therefore, for this objective, we focused on the direction and strength of the association between usage and physical activity. There were 18 studies reporting a usage outcome, of which 7 were excluded from the meta-analysis [22,24,45,46,52,54,56], as data were not available to allow the calculation of an effect estimate or a measure of variability of the effect estimate data were not available.

**Figure 2:**
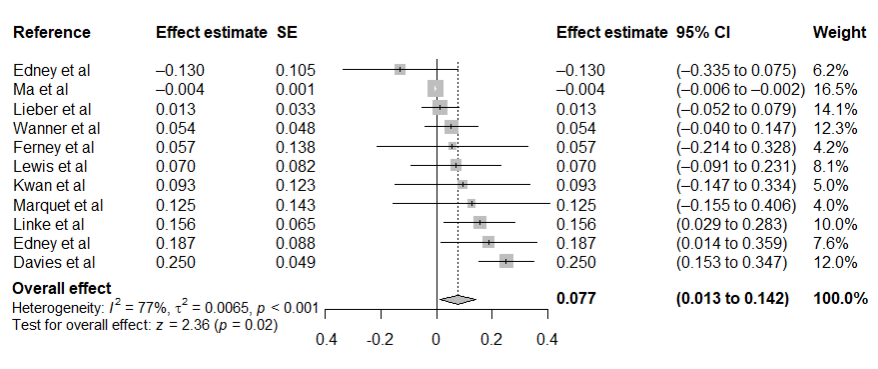
Meta-analysis results from 11 studies to assess the direction and strength of the relationship between engagement with a digital health intervention and physical activity using the Dersimonian and Laird method.

The results from the meta-analysis of usage associations (n=11 studies) are shown in Figure 2 [41-44,47-51,53,55]. The characteristics of each of the associations from the meta-analysis are included in Table 3. The pooled estimate of the standardized regression coefficient (0.08; 95% CI 0.01-0.14; *P*=.02; SD 0.11) indicated a small but significant positive relationship between engagement with a DHI and physical activity. Heterogeneity was high, with 77% of the variation in the point estimates explained by the between-study heterogeneity.

**Table 3:** Characteristics of studies (n=11) included in meta-analysis^a^.

Study	DHI^b^ type	Engagement measure	Physical activity measure
Edney et al [42]	Facebook	Activities completed	MVPA^c^
Ma et al [51]	Smartphone app	Time	Distance travelled
Lieber et al [49]	Web-based	Activities completed	MVPA
Wanner et al [55]	Web-based	Time	MVPA
Ferney et al [44]	Web-based	Logins	MVPA
Lewis et al [48]	Web-based	Activities completed	MVPA
Kwan et al [47]	Web-based	Logins	MVPA
Marquet et al [53]	Smartphone app	Time	Steps
Linke et al [50]	Web-based	Time	MVPA
Edney et al [43]	Smartphone app	Activities completed	MVPA
Davies et al [41]	Web-based	Activities completed	Steps

^a^A single association was selected for each study based on hierarchical criteria, see methods section. See Multimedia Appendix 5 for full details.

^b^DHI: digital health intervention.

^c^MVPA: moderate to vigorous physical activity.

The findings of the 7 studies that could not be included in the meta-analysis are summarized in Table 4. Four of the 7 studies reported an association consistent with the hypothesis that higher engagement (usage measures) is associated with a higher physical activity [22,45,52,56]. The remaining 3 studies had inconclusive findings [24,46,54]. Two studies had focused on the activities completed [24,56], one on time [54], and the remaining 4 on logins [22,45,46,52]. The inconclusive findings [24,46,54] were from the associations of different constructs of engagement (ie, activities completed, time, and logins).

**Table 4:** Characteristics and vote counting of studies (n=7) not included in meta-analysis.

Study	DHI^a^ type	Engagement measure	Physical activity measure	Association type	Association	Direction^b^
Carr et al [24]	Web-based	Activities completed	Steps	Multiple linear regression	Text only: “No other predictors,...[including engagement]...significantly explained...change in physical activity.”	0
Hansen et al [45]	Web-based	Logins	MVPA^c^	Kruskal-Wallis test	*P*≤.001	+
Hoj et al [46]	Smartphone app	Logins	Physical activity score	Multiple regression	SE −0.01 (0.067)	0
Maher et al [52]	Web-based	Logins	MVPA	Generalized linear mixed models	(F_1,41_=3.06; *P*=.04)	+
Marcus et al [22]	Web-based	Logins	MVPA	Quantile regression	β=34.32 (95% CI 14.33 to 54.31)	+
Rebar et al [54]	Web-based	Time	MVPA	Linear mixed models	γ=0.51 (95% CI −1.77 to 2.72); *P*>.05	0
Xian et al [56]	Smartphone app	Activities completed	Steps	Ordinal least squares regression	Every 10,000 XP points gained were associated with 2134 additional steps per day (95% CI 1673 to 2595; *P*<.001; R^2^=0.33])	+

^a^DHI: digital health intervention.

^b^A single association was selected for each study based on hierarchical criteria, see Methods section. Each study was summarized as either “+,” “−,” or “0.” “+” was assigned to the studies in which the point estimate and CI supported the hypothesis that higher engagement is associated with higher physical activity or reduced sedentary behavior. “0” was assigned to studies in which the point estimate and CI had inconclusive findings. “−“ was assigned to studies where the association point estimate and CI rejected the hypothesis. We assigned either “+” or “−“ to the studies without point estimates or CIs that reported significant association findings. We assigned “0” to the studies without point estimates or CIs that reported nonsignificant findings. See Multimedia Appendix 5 and Methods section for full details.

^c^MVPA: moderate to vigorous physical activity.

### Objective 2

Explore whether the direction of association between engagement and physical activity or sedentary behavior varies with the type of engagement (ie, subjective experience, activities completed, time, and logins).

Studies measured associations between physical activity and subjective experience (n=3) [40,46,48], activities completed (n=8) [24,41-43,48-50,56], time (n=5) [50,51,53-55], and logins (n=10) [22,24,44-48,50,52,54]. Therefore, 26 measures of association were included. The results of vote counting are summarized in Table 5. Overall, most associations (15 of 26) were in the hypothesized direction, stating that higher engagement is associated with higher physical activity. One association rejected the hypothesized direction, and the remaining 10 associations had inconclusive findings.

For the three domains of engagement, the direction consistently supported the hypothesis: subjective experience (2 of 3) [46,48], activities completed (5 of 8) [43,48-50,56], and logins (6 of 10) [22,45,48,50,52,54]. However, for time (n=5 associations), the findings did not support a positive association consistently, 2 studies had inconclusive findings [54,55], and one association rejected the hypothesized direction [51].

The 3 studies that described an association between subjective experience and physical activity used different measures to assess subjective experience. Bronner et al [40] used an Exergame Questionnaire that included questions similar to previously validated questions to assess subjective experience engagement in video games. The Exergame Questionnaire contained separate sections for engagement, game flow, and usability. The length of the questionnaire was not clear. Hoj et al [46] devised a five-question subjective experience questionnaire and constructed a composite score from it. Finally, Lewis et al [48] used the five-item Website Quality Questionnaire and constructed a composite score.

**Table 5:** Summary of associations included in vote counting.

Study	Engagement measure	DHI^a^ type	Association type	Association	Direction^b^
Bronner et al [40]	Subjective experience	Exergame	Pearson’s correlation	3 Associations: ρ=0.61 ρ=0.52 Not reported	0
Hoj et al [46]	Subjective experience	Smartphone app	Multiple regression	SE 0.40 (0.074)	+
Lewis et al [48]	Subjective experience	Web-based	Quintile regression	*t*=2.32 (*P*≤.01)	+
Carr et al [24]	Activities completed	Web-based	Multiple regression	Not reported (nonsignificant)	0
Davies et al [41]	Activities completed	Web-based	Odds ratio	3 Associations: OR^c^ 2.80 (95% CI 1.45 to 5.40) Not reported (nonsignificant) Not reported (nonsignificant)	0
Edney et al [42]	Activities completed	Facebook group	Pearson’s correlation	ρ=−0.13	0
Edney et al [43]	Activities completed	Smartphone app	Linear mixed models	F_1,272_=4.5 (*P*=.04)	+
Lewis et al [48]	Activities completed	Web-based	Odds ratio	OR 1.29 (95% CI 1.14 to 1.47)	+
Lieber et al [49]	Activities completed	Web-based	Odds ratio	OR 1.05 (95% CI 1.01 to 1.09)	+
Linke et al [50]	Activities completed	Web-based	Generalized linear models	3 Associations: β=2.85; SE: 1.38 (*P*=.04) β=1.00; SE: 0.82 (*P*=.05) β=3.49; SE: 1.28 (*P*=.01)	+
Xian et al [56]	Activities completed	Smartphone app	Ordinal least squares regression	Every 10,000 XP were associated with 2134 additional steps per day (95% CI 1673 to 2595; *P*<.001; R^2^=0.33)	+
Linke et al [50]	Time	Web-based	Generalized linear models	β=0.48, SE: 0.20; *P*=.02	+
Ma et al [51]	Time	Smartphone app	Multi-level modelling	β=−0.005; *P*≤.001	−
Marquet et al [53]	Time	Smartphone app	ANCOVA	ρ=0.176; *P*<.05	+
Rebar et al [54]	Time	Web-based	Linear mixed models	2 associations: γ=2.33 (95% CI 0.09 to 4.64); *P*<.05 γ=0.51 (95% CI −1.77 to 2.72); *P*>.05	0
Wanner et al [55]	Time	Web-based	Linear regression	95% CI 0.58 (−0.43 to 1.59; *P*=.26)	0
Carr et al [24]	Logins	Web-based	Multiple regression	Not reported (nonsignificant)	0
Ferney et al [44]	Logins	Web-based	ANCOVA	4 Associations *P*=.69 *P*=.70 *P*=.09 *P*=.05	0
Hansen et al [45]	Logins	Web-based	Kruskal-Wallis test	*P*≤.001	+
Hoj et al [46]	Logins	Smartphone app	Multiple regression	SE −0.01 (0.067)	0
Kwan et al [47]	Logins	Web-based	ANOVA^d^	F_1,63_=1.54, *P*=.22, n_p_^2^=0.03	0
Lewis et al [48]	Logins	Web-based	Quintile regression	*t*=3.39 (*P*≤.01)	+
Linke et al [50]	Logins	Web-based	Generalized linear models	Not reported (nonsignificant)	+
Maher et al [52]	Logins	Web-based	Generalized linear mixed models	F_1,41_=3.06 (*P*=.04)	+
Marcus et al [22]	Logins	Web-based	Quantile regression	β=34.32 (95% CI 14.33 to 54.31)	+
Rebar et al [54]	Logins	Web-based	Linear mixed models	2 Associations: γ=3.18 (95% CI 1.15 to 5.07); *P*<.05 γ=2.04 (95% CI 0.29 to 3.84); *P*<.05	+

^a^DHI: digital health intervention.

^b^”+,” “−,” or “0” were assigned. “+” was assigned to studies where all associations within the particular engagement domain (subjective experience, activities completed, time and logins) where the point estimates and CIs supported the hypothesis that higher engagement is associated with higher physical activity or reduced sedentary behavior. “0” was assigned to the studies with inconclusive or mixed associations. “−“ was assigned to the studies where all point estimates and CIs rejected the hypothesis that higher engagement is associated with higher physical activity or reduced sedentary behavior. See Multimedia Appendix 5 and Methods section for full details.

^c^OR: odds ratio.

^d^ANOVA: analysis of variance.

## Discussion

### Principal Findings

The findings of this review suggest that there is a positive relationship between engagement with a physical activity, both objective usage and subjective experience, and physical activity outcomes in adults. The strength of the relationship between DHI usage and physical activity based on a meta-analysis of 11 studies is weak (0.08, 95% CI 0.01-0.14). The direction of the association between physical activity and engagement was consistent across different measures of engagement, including two measures of usage (activities completed and logins) and subjective experience, but was less clear for the third measure of usage—time (ie, session duration). The majority of associations for subjective experience, activities completed, and logins were positive, whereas the remainder were inconclusive. There was a mixture of positive, inconclusive, and a negative association. No studies have examined the relationship between DHI engagement and sedentary behavior outcomes.

### Findings in Context

This review updates by 10 years and expands on the review by Donkin et al [8], which identified 3 studies assessing the association between the usage of physical activity DHIs and physical activity outcomes [8]. In agreement with our review, Donkin et al [8] reported a consistent positive relationship between usage outcomes (eg, logins and activities completed) and DHIs targeting physical health (ie, psychological health, dietary behavior, physical activity, weight management, and smoking; 31/33 studies) [8]. Logins and activities completed were the most common engagement outcomes included in both the reviews. Donkin et al [8] did not include any studies with associations between time and physical health behavior, whereas our review contributes 5 studies [50,51,53-55]. Our findings for time were inconsistent, which aligns with the nonhealth studies exploring user engagement with internet-based news websites, which have found that time is not a reliable indicator of engagement [57].

We found a positive but weak relationship between DHI usage and physical activity. In contrast to the usage, it has been suggested that a clearer dose-response relationship exists between subjective experience engagement (eg, how captivating of attention a DHI is, the emotions a DHI elicits, and how interesting participants find a DHI) and effectiveness [20]. This further highlights the importance of defining the types of engagement outcomes as well as using multiple indicators of engagement when trying to understand the relationship between engagement variables and the effectiveness of DHIs [58,59].

This is the first review to examine the relationship between DHI subjective experience engagement and physical activity. Only 3 studies have reported associations between subjective experience and physical activity, with 2 of the associations in the hypothesized direction [40,46,48]. Each study used different self-reporting tools to assess different constructs of subjective experience (ie, website usefulness, app likeability, engagement, game-flow, and usability), which has previously been identified as an issue in the assessment of subjective experience engagement [60]. Such heterogeneity in constructs makes comparisons difficult, even though the direction of association is consistently positive. Future studies should focus on using consistent measures of subjective experience that are valid and reliable to enable comparisons between studies [61,62].

Further research into the relationship between subjective experience engagement and usage engagement is also warranted, as some qualitative studies suggest that usage is positively related to subjective experience [63,64]. For example, results from interviews with participants involved in an internet-based physical activity intervention reported that usage was positively influenced by subjective experience factors (eg, trust, reliability, and functionality of the program) [64]. Another study found that the sustained use of a Fitbit activity tracker was influenced by subjective experience–related factors (eg, empty batteries, broken trackers, and user experience) [63]. In other health behaviors, people who smoke and consume alcohol who wish to quit or cut down have suggested that the look, feel, app store quality rating, branding, and wording of the title are important while choosing or not choosing to use an app [65]. Therefore, improving subjective experience could increase the strength of the positive relationship between usage and physical activity outcomes found in this review.

### Strengths

A key strength of this review was the focus on two health behaviors (physical activity and sedentary behavior), reducing the heterogeneity and increasing the validity of findings [20]. This is the first review to examine the association between the subjective experience of DHI and health behavior. Including subjective experience and usage recognizes that engagement goes beyond usage, while considering attention, interest, and affect [20]. Excluding DHIs targeting individuals with a specific health condition reduces heterogeneity, as the context (including population) is known to influence engagement [20]. Another strength is the use of meta-analyses to examine the strength of the association between DHI usage and physical activity. Although it was not possible to conduct separate meta-analyses within engagement constructs, the use of vote-counting methods to assess the direction of association is a recommended method when meta-analyses are not possible [39].

### Limitations

This review should be interpreted in the context of its limitations. The first limitation was that we included many different study types that produced large heterogeneity in the included studies. It means that we had to transform effect estimates to a common effect estimate and combine standardized effects, making interpretation of the results difficult. Furthermore, 8 studies were excluded from our meta-analysis because they provided insufficient information to be included in the meta-analysis. For vote counting, where studies did not report point estimates and CIs, we had to rely on the wording provided by the authors to infer whether the results supported our hypothesis. Second, the analysis of the association between engagement outcomes and physical activity outcomes in all 19 studies was a secondary analysis for these studies. Such analyses were often not well described. A further limitation was the inclusion of all recruitment types (ie, ecological and nonecological) within the same meta-analysis and vote-counting (ie, ecological and nonecological). It is known that ecological recruitment methods lead to higher attrition and lower engagement [31]. For example, Wanner et al [55] and Vandelanotte et al [31] highlight that spontaneous users (ecological) report a much lower engagement and higher dropout, whereas those that remain engaged become as active as those in the randomized groups (nonecological groups), possibly due to differing motivations [31,55]. In addition, although our search methods were rigorous, it is possible that expanding the search databases to include the ACM Digital Library may have identified additional studies from the human-computer interaction literature. Finally, given the lack of quantitative studies on subjective experience, perhaps owing to subjective experience being more often measured qualitatively [20], we encourage future reviews to explore the relationship between engagement and physical activity and sedentary behavior in qualitative studies.

### Conclusions

A weak but consistent positive relationship exists between engagement with a physical activity DHI and physical activity outcomes. This is consistent across 2 of the 3 indicators of usage engagement that we examined, and subjective experience engagement; however, there are weak effect sizes. A further exploration of the relationship between engagement and physical activity using valid and reliable measurement tools is warranted, given the heterogeneity in measurement tools. Additional focus should be directed at DHI subjective experience (ie, attention, interest, and affect) by using consistent methodology to explore its relationship with the usage of DHIs and health behavior outcomes. Given the absence of studies, further research examining the association between DHIs and the impact on sedentary behavior is also warranted.

## Appendix

Multimedia Appendix 1 Search strategy.

Multimedia Appendix 2 Newcastle Ottawa Scale for cohort studies.

Multimedia Appendix 3 Newcastle Ottawa Scale for cross-sectional studies.

Multimedia Appendix 4 Data transformations.

Multimedia Appendix 5 Supplementary table of characteristics of included studies.

## Abbreviations

DHI: digital health intervention
PRISMA: Preferred Reporting Items for Systematic Reviews and Meta-Analyses
PROSPERO: International Prospective Register for Systematic Reviews
