# Correction: Associations Between Digital Health Intervention Engagement, Physical Activity, and Sedentary Behavior: Systematic Review and Meta-analysis

**DOI:** 10.2196/29094

**Published:** 2021-03-29

**Authors:** Matthew Mclaughlin, Tessa Delaney, Alix Hall, Judith Byaruhanga, Paul Mackie, Alice Grady, Kathryn Reilly, Elizabeth Campbell, Rachel Sutherland, John Wiggers, Luke Wolfenden

**Affiliations:** 1 School of Medicine and Public Health University of Newcastle Callaghan Australia; 2 Hunter New England Population Health Wallsend Australia; 3 Hunter Medical Research Institute New Lambton Heights Australia; 4 Priority Research Centre for Heath Behaviour University of Newcastle Callaghan Australia; 5 School of Health Sciences and Priority Research Centre for Stroke and Brain Injury, University of Newcastle Callaghan Australia; 6 Centre for Research Excellence in Stroke Recovery and Rehabilitation, Florey Institute of Neuroscience Melbourne Australia

In “Associations Between Digital Health Intervention Engagement, Physical Activity, and Sedentary Behavior: Systematic Review and Meta-analysis” (J Med Internet Res 2021;23(2):e23180) a character display error was noted in 3 tables.

The “gamma” symbol (γ) was not properly rendered in 5 places in the paper due to an XML conversion error.

In Table 4, row “Rebar et al,” column “Association”:

=0.51 (95% CI −1.77 to 2.72); *P*>.05

has been corrected to:

γ=0.51 (95% CI −1.77 to 2.72); *P*>.05

In Table 5, row “Rebar et al, Time,” column “Association”:

=2.33 (95% CI 0.09 to 4.64); *P*<.05

has been corrected to:

γ=2.33 (95% CI 0.09 to 4.64); *P*<.05

and:

=0.51 (95% CI −1.77 to 2.72); *P*>.05

has been corrected to:

γ=0.51 (95% CI −1.77 to 2.72); *P*>.05

In Table 5, row “Rebar et al, Logins,” column “Association”:

=3.18 (95% CI 1.15 to 5.07); *P*<.05

has been corrected to:

γ=3.18 (95% CI 1.15 to 5.07); *P*<.05

and:

=2.04 (95% CI 0.29 to 3.84); *P*<.05

has been corrected to:

γ=2.04 (95% CI 0.29 to 3.84); *P*<.05

The correction will appear in the online version of the paper on the JMIR Publications website on March 29, 2021, together with the publication of this correction notice. Because this was made after submission to PubMed, PubMed Central, and other full-text repositories, the corrected article has also been resubmitted to those repositories.

