# Supplementary material for: Associations Between Digital Health Intervention Engagement, Physical Activity, and Sedentary Behavior: Systematic Review and Meta-analysis
Source: J Med Internet Res. 2021 Feb 19;23(2):e23180. doi: 10.2196/23180 (PMC8011420; doi:10.2196/23180)
Supplement: Multimedia Appendix 1 [file jmir_v23i2e23180_app1.docx]

**Multimedia Appendix 1**

Searches conducted: 4/9/18 and 5/9/18 AND then updated 5/12/2019.

| **Database** | **Records identified** | **Records after de-duplication** |
| --- | --- | --- |
| Academic Search Ultimate | 2287 | 710 |
| CENTRAL | 2844 | 1882 |
| CINAHL | 965 | 321 |
| Embase | 2621 | 768 |
| Medline | 1805 | 414 |
| PsycINFO | 897 | 146 |
| Scopus | 1773 | 691 |
| **TOTAL** | **13,192** | **4932** |

Database(s): **Ovid MEDLINE(R) and Epub Ahead of Print, In-Process & Other Non-Indexed Citations and Daily**1946 to August 31, 2018 
Search Strategy:

| **#** | **Searches** |
| --- | --- |
| 1 | exercise/ or gymnastics/ or physical conditioning, human/ or running/ or swimming/ or walking/ |
| 2 | Motor Activity/ |
| 3 | (physical education and training).mp. |
| 4 | physical fitness/ or cardiorespiratory fitness/ |
| 5 | life style/ or healthy lifestyle/ or healthy aging/ or healthy diet/ or sedentary lifestyle/ |
| 6 | exp sports/ |
| 7 | physical* inactiv*.ti,ab,kw. |
| 8 | physical* activ*.ti,ab,kw. |
| 9 | sedentary.ti,ab,kw. |
| 10 | Exercis*.ti,ab,kw. |
| 11 | Sport*.ti,ab,kw. |
| 12 | (("lifestyle*" or life style*) adj5 activ*).ti,ab,kw. |
| 13 | (fitness adj (class* or regime* or program*)).mp. |
| 14 | 1 or 2 or 3 or 4 or 5 or 6 or 7 or 8 or 9 or 10 or 11 or 12 or 13 |
| 15 | exp Diet/ |
| 16 | nutrition*.ti,ab,kw. |
| 17 | Healthy eating.ti,ab,kw. |
| 18 | Child nutrition sciences/ |
| 19 | fruit*.ti,ab,kw. |
| 20 | Fruit/ |
| 21 | Vegetable*.ti,ab,kw. |
| 22 | Vegetables/ |
| 23 | Canteen*.ti,ab,kw. |
| 24 | Food services/ or food service*.ti,ab,kw. |
| 25 | Menu planning/ or menu*.ti,ab,kw. |
| 26 | calorie*.ti,ab,kw. |
| 27 | Energy intake/ |
| 28 | energy density.ti,ab,kw. |
| 29 | eating/ |
| 30 | Feeding Behavior/ or feeding behavio*.ti,ab,kw. |
| 31 | dietary intake.ti,ab,kw. |
| 32 | food habits.ti,ab,kw. |
| 33 | food/ |
| 34 | Carbonated Beverages/ or soft drink*.ti,ab,kw. |
| 35 | soda.ti,ab,kw. |
| 36 | sweetened drink*.ti,ab,kw. |
| 37 | Dietary Fats, Unsaturated/ or Dietary Fats/ |
| 38 | confectionar*.ti,ab,kw. |
| 39 | (school adj (lunch* or meal*)).ti,ab,kw. |
| 40 | ((feeding or food or nutrition*) adj program*).ti,ab,kw. |
| 41 | cafeteria*.ti,ab,kw. |
| 42 | (food adj2 (purchas* or select*)).ti,ab,kw. |
| 43 | 15 or 16 or 17 or 18 or 19 or 20 or 21 or 22 or 23 or 24 or 25 or 26 or 27 or 28 or 29 or 30 or 31 or 32 or 33 or 34 or 35 or 36 or 37 or 38 or 39 or 40 or 41 or 42 |
| 44 | System* usability scale.ti,ab,kw. |
| 45 | ((intervention or app or e-mail or email or m-health or e-health or digital health or online or web*) adj5 (adopt* or uptake or retention or maintenance or participat* or engag* or usage attrition or usage or adher*)).ti,ab,kw. |
| 46 | (process adj (metric* or evaluation)).ti,ab,kw. |
| 47 | login*.ti,ab,kw. |
| 48 | (page* adj3 view*).ti,ab,kw. |
| 49 | ((module* or session*) adj complet*).ti,ab,kw. |
| 50 | (visit* adj3 website*).ti,ab,kw. |
| 51 | (time adj3 online).ti,ab,kw. |
| 52 | (user adj3 (engag* or experience* or enjoyment or attention or interest or affect or immersion or involvement)).ti,ab,kw. |
| 53 | usability.ti,ab,kw. |
| 54 | 44 or 45 or 46 or 47 or 48 or 49 or 50 or 51 or 52 or 53 |
| 55 | Smartphone/ or Cell Phone/ or mobile device*.ti,ab,kw. or Mobile Applications/ |
| 56 | Virtual reality/ or (virtual adj reality).ti,ab,kw. |
| 57 | Online Systems/ or online.ti,ab,kw. |
| 58 | Internet.ti,ab,kw. |
| 59 | (web adj based).ti,ab,kw. |
| 60 | (world wide web or WWW or website*).ti,ab,kw. |
| 61 | Electronic Mail/ or (e-mail* or email*).ti,ab,kw. |
| 62 | ((mobile or cellular or cell or smart) adj (phone* or telephone*)).ti,ab,kw. |
| 63 | (e-health or ehealth or electronic health).ti,ab,kw. |
| 64 | (m-health or mhealth or mobile health).ti,ab,kw. |
| 65 | ((digital health or digital) adj3 intervention*).ti,ab,kw. |
| 66 | (interactive adj ((health adj communicat*) or video* or technolog* or multimedia)).ti,ab,kw. |
| 67 | ((chat adj room*) or chatroom*).ti,ab,kw. |
| 68 | (phone adj app*).ti,ab,kw. |
| 69 | User-Computer Interface/ or human computer interaction*.ti,ab,kw. |
| 70 | 55 or 56 or 57 or 58 or 59 or 60 or 61 or 62 or 63 or 64 or 65 or 66 or 67 or 68 or 69 |
| 71 | 14 or 43 |
| 72 | 54 and 70 and 71 |
| 73 | animals/ not (humans/ and animals/) |
| 74 | 72 not 73 |
| **75** | **limit 74 to english language** |

Database(s): **Embase**1947 to present 
Search Strategy:

| **#** | **Searches** |
| --- | --- |
| 1 | exercise/ |
| 2 | physical education/ |
| 3 | running/ |
| 4 | swimming/ |
| 5 | walking/ |
| 6 | motor activity/ |
| 7 | fitness/ |
| 8 | cardiorespiratory fitness/ |
| 9 | lifestyle/ or healthy lifestyle/ or sedentary lifestyle/ |
| 10 | healthy aging/ |
| 11 | healthy diet/ |
| 12 | exp sport/ |
| 13 | physical activity/ or physical inactivity/ |
| 14 | (physical* inactiv* or physical* activ*).ti,ab,kw. |
| 15 | sedentary.ti,ab,kw. |
| 16 | Exercis*.ti,ab,kw. |
| 17 | Sport*.ti,ab,kw. |
| 18 | (("lifestyle*" or life style*) adj5 activ*).ti,ab,kw. |
| 19 | (fitness adj (class* or regime* or program*)).mp. |
| 20 | (gymnastic* or physical conditioning).ti,ab,kw. |
| 21 | 1 or 2 or 3 or 4 or 5 or 6 or 7 or 8 or 9 or 10 or 11 or 12 or 13 or 14 or 15 or 16 or 17 or 18 or 19 or 20 |
| 22 | exp diet/ |
| 23 | nutrition*.ti,ab,kw. |
| 24 | Healthy eating.ti,ab,kw. |
| 25 | Child nutrition sciences.ti,ab,kw. |
| 26 | fruit*.ti,ab,kw. |
| 27 | fruit/ |
| 28 | Vegetable*.ti,ab,kw. |
| 29 | vegetable/ |
| 30 | Canteen*.ti,ab,kw. |
| 31 | catering service/ |
| 32 | food service*.ti,ab,kw. |
| 33 | menu*.ti,ab,kw. |
| 34 | calorie*.ti,ab,kw. |
| 35 | caloric intake/ |
| 36 | energy density.ti,ab,kw. |
| 37 | eating/ |
| 38 | Feeding Behavior/ or feeding behavio*.ti,ab,kw. |
| 39 | dietary intake.ti,ab,kw. |
| 40 | food habits.ti,ab,kw. |
| 41 | food/ |
| 42 | Carbonated Beverage/ or soft drink*.ti,ab,kw. |
| 43 | soda.ti,ab,kw. |
| 44 | sweetened drink*.ti,ab,kw. |
| 45 | fat intake/ or dietary intake/ |
| 46 | confectionar*.ti,ab,kw. |
| 47 | (school adj (lunch* or meal*)).ti,ab,kw. |
| 48 | ((feeding or food or nutrition*) adj program*).ti,ab,kw. |
| 49 | cafeteria*.ti,ab,kw. |
| 50 | (food adj2 (purchas* or select*)).ti,ab,kw. |
| 51 | 22 or 23 or 24 or 25 or 26 or 27 or 28 or 29 or 30 or 31 or 32 or 33 or 34 or 35 or 36 or 37 or 38 or 39 or 40 or 41 or 42 or 43 or 44 or 45 or 46 or 47 or 48 or 49 or 50 |
| 52 | System* usability scale.ti,ab,kw. |
| 53 | ((intervention or app or e-mail or email or m-health or e-health or digital health or online or web*) adj5 (adopt* or uptake or retention or maintenance or participat* or engag* or usage attrition or usage or adher*)).ti,ab,kw. |
| 54 | (process adj (metric* or evaluation)).ti,ab,kw. |
| 55 | login*.ti,ab,kw. |
| 56 | (page* adj3 view*).ti,ab,kw. |
| 57 | ((module* or session*) adj complet*).ti,ab,kw. |
| 58 | (visit* adj3 website*).ti,ab,kw. |
| 59 | (time adj3 online).ti,ab,kw. |
| 60 | (user adj3 (engag* or experience* or enjoyment or attention or interest or affect or immersion or involvement)).ti,ab,kw. |
| 61 | usability.ti,ab,kw. |
| 62 | 52 or 53 or 54 or 55 or 56 or 57 or 58 or 59 or 60 or 61 |
| 63 | smartphone/ or mobile phone/ |
| 64 | mobile application/ |
| 65 | mobile device*.ti,ab,kw. |
| 66 | Virtual reality/ or (virtual adj reality).ti,ab,kw. |
| 67 | Online System/ or online.ti,ab,kw. |
| 68 | Internet/ or Internet.ti,ab,kw. |
| 69 | (web adj based).ti,ab,kw. |
| 70 | (world wide web or WWW or website*).ti,ab,kw. |
| 71 | e-Mail/ or (e-mail* or email* or electronic mail).ti,ab,kw. |
| 72 | ((mobile or cellular or cell or smart) adj (phone* or telephone*)).ti,ab,kw. |
| 73 | (e-health or ehealth or electronic health).ti,ab,kw. |
| 74 | (m-health or mhealth or mobile health).ti,ab,kw. |
| 75 | ((digital health or digital) adj3 intervention*).ti,ab,kw. |
| 76 | (interactive adj ((health adj communicat*) or video* or technolog* or multimedia)).ti,ab,kw. |
| 77 | ((chat adj room*) or chatroom*).ti,ab,kw. |
| 78 | (phone adj app*).ti,ab,kw. |
| 79 | Computer Interface/ or human computer interaction*.ti,ab,kw. |
| 80 | 63 or 64 or 65 or 66 or 67 or 68 or 69 or 70 or 71 or 72 or 73 or 74 or 75 or 76 or 77 or 78 or 79 |
| 81 | 21 or 51 |
| 82 | 62 and 80 and 81 |
| 83 | animal/ not (human/ and animal/) |
| 84 | 82 not 83 |
| 85 | limit 84 to english language |

Database(s): **PsycINFO**1806 to August Week 4 2018 
Search Strategy:

| **#** | **Searches** |
| --- | --- |
| 1 | exercise/ or physical activity/ or physical fitness/ |
| 2 | exp sports/ |
| 3 | lifestyle/ or active living/ or lifestyle changes/ |
| 4 | (physical education and training).mp. |
| 5 | (gymnastics or physical conditioning or running or walking).ti,ab,id. |
| 6 | motor activity.ti,ab,id. |
| 7 | (physical education and training).ti,ab,id. |
| 8 | (physical activ* or physical* inactiv*).ti,ab,id. |
| 9 | (sedentary or exercis* or sport* or healthy diet or healthy ag?ing).ti,ab,id. |
| 10 | (("lifestyle*" or life style*) adj5 activ*).ti,ab,id. |
| 11 | (fitness adj (class* or regime* or program*)).ti,ab,id. |
| 12 | 1 or 2 or 3 or 4 or 5 or 6 or 7 or 8 or 9 or 10 or 11 |
| 13 | diets/ |
| 14 | nutrition*.ti,ab,id. |
| 15 | Healthy eating.ti,ab,id. |
| 16 | (fruit* or vegetable*).ti,ab,id. |
| 17 | (canteen* or food service* or menu* or calorie*).ti,ab,id. |
| 18 | Food Intake/ |
| 19 | Eating Behavior/ |
| 20 | "beverages (nonalcoholic)"/ |
| 21 | FOOD/ |
| 22 | (energy intake or energy density or feeding behavio* or dietary intake or food habits or carbonated beverage* or soft drink* or soda or sweetened drink* or dietary fat* or confectionar*).ti,ab,id. |
| 23 | (school adj (lunch* or meal*)).ti,ab,id. |
| 24 | ((feeding or food or nutrition*) adj program*).ti,ab,id. |
| 25 | cafeteria*.ti,ab,id. |
| 26 | (food adj2 (purchas* or select*)).ti,ab,id. |
| 27 | 13 or 14 or 15 or 16 or 17 or 18 or 19 or 20 or 21 or 22 or 23 or 24 or 25 or 26 |
| 28 | System* usability scale.ti,ab,id. |
| 29 | ((intervention or app or e-mail or email or m-health or e-health or digital health or online or web*) adj5 (adopt* or uptake or retention or maintenance or participat* or engag* or usage attrition or usage or adher*)).ti,ab,id. |
| 30 | (process adj (metric* or evaluation)).ti,ab,id. |
| 31 | login*.ti,ab,id. |
| 32 | (page* adj3 view*).ti,ab,id. |
| 33 | ((module* or session*) adj complet*).ti,ab,id. |
| 34 | (visit* adj3 website*).ti,ab,id. |
| 35 | (time adj3 online).ti,ab,id. |
| 36 | (user adj3 (engag* or experience* or enjoyment or attention or interest or affect or immersion or involvement)).ti,ab,id. |
| 37 | usability.ti,ab,id. |
| 38 | 28 or 29 or 30 or 31 or 32 or 33 or 34 or 35 or 36 or 37 |
| 39 | exp Cellular Phones/ or exp Mobile Devices/ |
| 40 | mobile device*.ti,ab,id. |
| 41 | Virtual reality/ or (virtual adj reality).ti,ab,id. |
| 42 | online.ti,ab,id. |
| 43 | Internet/ or Internet.ti,ab,id. |
| 44 | (web adj based).ti,ab,id. |
| 45 | (world wide web or WWW or website*).ti,ab,id. |
| 46 | Computer Mediated Communication/ or (e-mail* or email* or electronic mail).ti,ab,id. |
| 47 | ((mobile or cellular or cell or smart) adj (phone* or telephone*)).ti,ab,id. |
| 48 | (e-health or ehealth or electronic health).ti,ab,id. |
| 49 | (m-health or mhealth or mobile health).ti,ab,id. |
| 50 | ((digital health or digital) adj3 intervention*).ti,ab,id. |
| 51 | (interactive adj ((health adj communicat*) or video* or technolog* or multimedia)).ti,ab,id. |
| 52 | ((chat adj room*) or chatroom*).ti,ab,id. |
| 53 | (phone adj app*).ti,ab,id. |
| 54 | Human Computer Interface/ or human computer interaction*.ti,ab,id. |
| 55 | 39 or 40 or 41 or 42 or 43 or 44 or 45 or 46 or 47 or 48 or 49 or 50 or 51 or 52 or 53 or 54 |
| 56 | 12 or 27 |
| 57 | 38 and 55 and 56 |
| 58 | limit 57 to (human and english language) |

CINAHL

| **#** | **Query** | **Limiters/Expanders** | **Last Run Via** | **Results** |
| --- | --- | --- | --- | --- |
| S1 | (MH "Exercise") | Search modes - Boolean/Phrase | Interface - EBSCOhost Research Databases  Search Screen - Advanced Search  Database - CINAHL Complete | 41,716 |
| S2 | (MH "Gymnastics") | Search modes - Boolean/Phrase | Interface - EBSCOhost Research Databases  Search Screen - Advanced Search  Database - CINAHL Complete | 761 |
| S3 | (MH "Running") | Search modes - Boolean/Phrase | Interface - EBSCOhost Research Databases  Search Screen - Advanced Search  Database - CINAHL Complete | 8,397 |
| S4 | (MH "Swimming") | Search modes - Boolean/Phrase | Interface - EBSCOhost Research Databases  Search Screen - Advanced Search  Database - CINAHL Complete | 3,614 |
| S5 | (MH "Walking") | Search modes - Boolean/Phrase | Interface - EBSCOhost Research Databases  Search Screen - Advanced Search  Database - CINAHL Complete | 18,452 |
| S6 | (MH "Motor Activity") | Search modes - Boolean/Phrase | Interface - EBSCOhost Research Databases  Search Screen - Advanced Search  Database - CINAHL Complete | 10,566 |
| S7 | (MH "Physical Education and Training") | Search modes - Boolean/Phrase | Interface - EBSCOhost Research Databases  Search Screen - Advanced Search  Database - CINAHL Complete | 2,997 |
| S8 | (MH "Physical Fitness") | Search modes - Boolean/Phrase | Interface - EBSCOhost Research Databases  Search Screen - Advanced Search  Database - CINAHL Complete | 14,657 |
| S9 | (MH "Cardiorespiratory Fitness") | Search modes - Boolean/Phrase | Interface - EBSCOhost Research Databases  Search Screen - Advanced Search  Database - CINAHL Complete | 321 |
| S10 | (MH "Life Style") OR (MH "Life Style, Sedentary") | Search modes - Boolean/Phrase | Interface - EBSCOhost Research Databases  Search Screen - Advanced Search  Database - CINAHL Complete | 27,781 |
| S11 | "healthy aging" | Search modes - Boolean/Phrase | Interface - EBSCOhost Research Databases  Search Screen - Advanced Search  Database - CINAHL Complete | 1,427 |
| S12 | "healthy diet" | Search modes - Boolean/Phrase | Interface - EBSCOhost Research Databases  Search Screen - Advanced Search  Database - CINAHL Complete | 1,819 |
| S13 | (MH "Sports+") | Search modes - Boolean/Phrase | Interface - EBSCOhost Research Databases  Search Screen - Advanced Search  Database - CINAHL Complete | 66,952 |
| S14 | TI "physical* inactiv*" OR AB "physical* inactiv*" | Search modes - Boolean/Phrase | Interface - EBSCOhost Research Databases  Search Screen - Advanced Search  Database - CINAHL Complete | 3,119 |
| S15 | TI "physical* activ*" OR AB "physical* activ*" | Search modes - Boolean/Phrase | Interface - EBSCOhost Research Databases  Search Screen - Advanced Search  Database - CINAHL Complete | 47,212 |
| S16 | TI sedentary OR AB sedentary | Search modes - Boolean/Phrase | Interface - EBSCOhost Research Databases  Search Screen - Advanced Search  Database - CINAHL Complete | 9,062 |
| S17 | TI Exercis* OR AB Exercis* | Search modes - Boolean/Phrase | Interface - EBSCOhost Research Databases  Search Screen - Advanced Search  Database - CINAHL Complete | 94,069 |
| S18 | TI Sport* OR AB Sport* | Search modes - Boolean/Phrase | Interface - EBSCOhost Research Databases  Search Screen - Advanced Search  Database - CINAHL Complete | 33,004 |
| S19 | TI ( (("lifestyle*" or life style*) n5 activ*) ) OR AB ( (("lifestyle*" or life style*) n5 activ*) ) | Search modes - Boolean/Phrase | Interface - EBSCOhost Research Databases  Search Screen - Advanced Search  Database - CINAHL Complete | 2,897 |
| S20 | TI ( (fitness n1 (class* or regime* or program*)) ) OR AB ( (fitness n1 (class* or regime* or program*)) ) | Search modes - Boolean/Phrase | Interface - EBSCOhost Research Databases  Search Screen - Advanced Search  Database - CINAHL Complete | 699 |
| S21 | S1 OR S2 OR S3 OR S4 OR S5 OR S6 OR S7 OR S8 OR S9 OR S10 OR S11 OR S12 OR S13 OR S14 OR S15 OR S16 OR S17 OR S18 OR S19 OR S20 | Search modes - Boolean/Phrase | Interface - EBSCOhost Research Databases  Search Screen - Advanced Search  Database - CINAHL Complete | 261,276 |
| S22 | (MH "Diet+") | Search modes - Boolean/Phrase | Interface - EBSCOhost Research Databases  Search Screen - Advanced Search  Database - CINAHL Complete | 95,191 |
| S23 | (MH "Nutrition") OR "nutrition*" | Search modes - Boolean/Phrase | Interface - EBSCOhost Research Databases  Search Screen - Advanced Search  Database - CINAHL Complete | 126,356 |
| S24 | TI "Healthy eating" OR AB "Healthy eating" | Search modes - Boolean/Phrase | Interface - EBSCOhost Research Databases  Search Screen - Advanced Search  Database - CINAHL Complete | 4,140 |
| S25 | "Child nutrition sciences" | Search modes - Boolean/Phrase | Interface - EBSCOhost Research Databases  Search Screen - Advanced Search  Database - CINAHL Complete | 19,641 |
| S26 | TI fruit* OR AB fruit* | Search modes - Boolean/Phrase | Interface - EBSCOhost Research Databases  Search Screen - Advanced Search  Database - CINAHL Complete | 15,924 |
| S27 | (MH "Fruit") | Search modes - Boolean/Phrase | Interface - EBSCOhost Research Databases  Search Screen - Advanced Search  Database - CINAHL Complete | 10,847 |
| S28 | TI Vegetable* OR AB Vegetable* | Search modes - Boolean/Phrase | Interface - EBSCOhost Research Databases  Search Screen - Advanced Search  Database - CINAHL Complete | 12,241 |
| S29 | (MH "Vegetables") | Search modes - Boolean/Phrase | Interface - EBSCOhost Research Databases  Search Screen - Advanced Search  Database - CINAHL Complete | 10,264 |
| S30 | TI Canteen* OR AB Canteen* | Search modes - Boolean/Phrase | Interface - EBSCOhost Research Databases  Search Screen - Advanced Search  Database - CINAHL Complete | 210 |
| S31 | (MH "Food Services") OR "food service*" | Search modes - Boolean/Phrase | Interface - EBSCOhost Research Databases  Search Screen - Advanced Search  Database - CINAHL Complete | 6,919 |
| S32 | (MH "Menu Planning") OR "menu*" | Search modes - Boolean/Phrase | Interface - EBSCOhost Research Databases  Search Screen - Advanced Search  Database - CINAHL Complete | 2,971 |
| S33 | "calorie*" | Search modes - Boolean/Phrase | Interface - EBSCOhost Research Databases  Search Screen - Advanced Search  Database - CINAHL Complete | 5,939 |
| S34 | (MH "Energy Intake") | Search modes - Boolean/Phrase | Interface - EBSCOhost Research Databases  Search Screen - Advanced Search  Database - CINAHL Complete | 15,044 |
| S35 | (MH "Energy Density") | Search modes - Boolean/Phrase | Interface - EBSCOhost Research Databases  Search Screen - Advanced Search  Database - CINAHL Complete | 740 |
| S36 | (MH "Eating") | Search modes - Boolean/Phrase | Interface - EBSCOhost Research Databases  Search Screen - Advanced Search  Database - CINAHL Complete | 5,375 |
| S37 | (MH "Eating Behavior") | Search modes - Boolean/Phrase | Interface - EBSCOhost Research Databases  Search Screen - Advanced Search  Database - CINAHL Complete | 12,075 |
| S38 | TI dietary intake OR AB dietary intake | Search modes - Boolean/Phrase | Interface - EBSCOhost Research Databases  Search Screen - Advanced Search  Database - CINAHL Complete | 12,741 |
| S39 | (MH "Food Habits") | Search modes - Boolean/Phrase | Interface - EBSCOhost Research Databases  Search Screen - Advanced Search  Database - CINAHL Complete | 10,971 |
| S40 | (MH "Food") | Search modes - Boolean/Phrase | Interface - EBSCOhost Research Databases  Search Screen - Advanced Search  Database - CINAHL Complete | 12,209 |
| S41 | (MH "Carbonated Beverages") | Search modes - Boolean/Phrase | Interface - EBSCOhost Research Databases  Search Screen - Advanced Search  Database - CINAHL Complete | 2,150 |
| S42 | TI "soft drink*" OR AB "soft drink*" | Search modes - Boolean/Phrase | Interface - EBSCOhost Research Databases  Search Screen - Advanced Search  Database - CINAHL Complete | 1,123 |
| S43 | TI soda OR AB soda | Search modes - Boolean/Phrase | Interface - EBSCOhost Research Databases  Search Screen - Advanced Search  Database - CINAHL Complete | 954 |
| S44 | TI "sweetened drink*" OR AB "sweetened drink*" | Search modes - Boolean/Phrase | Interface - EBSCOhost Research Databases  Search Screen - Advanced Search  Database - CINAHL Complete | 151 |
| S45 | (MH "Dietary Fats") | Search modes - Boolean/Phrase | Interface - EBSCOhost Research Databases  Search Screen - Advanced Search  Database - CINAHL Complete | 11,184 |
| S46 | TI confectionar* OR AB confectionar* | Search modes - Boolean/Phrase | Interface - EBSCOhost Research Databases  Search Screen - Advanced Search  Database - CINAHL Complete | 57 |
| S47 | TI ( (school n1 (lunch* or meal*)) ) OR AB ( (school n1 (lunch* or meal*)) ) | Search modes - Boolean/Phrase | Interface - EBSCOhost Research Databases  Search Screen - Advanced Search  Database - CINAHL Complete | 941 |
| S48 | TI ( ((feeding or food or nutrition*) n1 program*) ) OR AB ( ((feeding or food or nutrition*) n1 program*) ) | Search modes - Boolean/Phrase | Interface - EBSCOhost Research Databases  Search Screen - Advanced Search  Database - CINAHL Complete | 3,376 |
| S49 | TI cafeteria* OR AB cafeteria* | Search modes - Boolean/Phrase | Interface - EBSCOhost Research Databases  Search Screen - Advanced Search  Database - CINAHL Complete | 511 |
| S50 | TI ( (food n2 (purchas* or select*)) ) OR AB ( (food n2 (purchas* or select*)) ) | Search modes - Boolean/Phrase | Interface - EBSCOhost Research Databases  Search Screen - Advanced Search  Database - CINAHL Complete | 1,709 |
| S51 | S22 OR S23 OR S24 OR S25 OR S26 OR S27 OR S28 OR S29 OR S30 OR S31 OR S32 OR S33 OR S34 OR S35 OR S36 OR S37 OR S38 OR S39 OR S40 OR S41 OR S42 OR S43 OR S44 OR S45 OR S46 OR S47 OR S48 OR S49 OR S50 | Search modes - Boolean/Phrase | Interface - EBSCOhost Research Databases  Search Screen - Advanced Search  Database - CINAHL Complete | 236,975 |
| S52 | TI ( ((intervention or app or e-mail or email or m-health or e-health or digital health or online or web*) n5 (adopt* or uptake or retention or maintenance or participat* or engag* or usage attrition or usage or adher*)) ) OR AB ( ((intervention or app or e-mail or email or m-health or e-health or digital health or online or web*) n5 (adopt* or uptake or retention or maintenance or participat* or engag* or usage attrition or usage or adher*)) ) | Search modes - Boolean/Phrase | Interface - EBSCOhost Research Databases  Search Screen - Advanced Search  Database - CINAHL Complete | 17,293 |
| S53 | TI ( (process n1 (metric* or evaluation)) ) OR AB ( (process n1 (metric* or evaluation)) ) | Search modes - Boolean/Phrase | Interface - EBSCOhost Research Databases  Search Screen - Advanced Search  Database - CINAHL Complete | 3,313 |
| S54 | TI login* OR AB login* | Search modes - Boolean/Phrase | Interface - EBSCOhost Research Databases  Search Screen - Advanced Search  Database - CINAHL Complete | 165 |
| S55 | TI (page* n3 view*) OR AB (page* n3 view*) | Search modes - Boolean/Phrase | Interface - EBSCOhost Research Databases  Search Screen - Advanced Search  Database - CINAHL Complete | 135 |
| S56 | TI ( ((module* or session*) n1 complet*) ) OR AB ( ((module* or session*) n1 complet*) ) | Search modes - Boolean/Phrase | Interface - EBSCOhost Research Databases  Search Screen - Advanced Search  Database - CINAHL Complete | 1,006 |
| S57 | TI (visit* n3 website*) OR AB (visit* n3 website*) | Search modes - Boolean/Phrase | Interface - EBSCOhost Research Databases  Search Screen - Advanced Search  Database - CINAHL Complete | 270 |
| S58 | TI (time n3 online) OR AB (time n3 online) | Search modes - Boolean/Phrase | Interface - EBSCOhost Research Databases  Search Screen - Advanced Search  Database - CINAHL Complete | 428 |
| S59 | TI ( (user n3 (engag* or experience* or enjoyment or attention or interest or affect or immersion or involvement)) ) OR AB ( (user n3 (engag* or experience* or enjoyment or attention or interest or affect or immersion or involvement)) ) | Search modes - Boolean/Phrase | Interface - EBSCOhost Research Databases  Search Screen - Advanced Search  Database - CINAHL Complete | 4,520 |
| S60 | TI usability OR AB usability | Search modes - Boolean/Phrase | Interface - EBSCOhost Research Databases  Search Screen - Advanced Search  Database - CINAHL Complete | 4,090 |
| S61 | S52 OR S53 OR S54 OR S55 OR S56 OR S57 OR S58 OR S59 OR S60 | Search modes - Boolean/Phrase | Interface - EBSCOhost Research Databases  Search Screen - Advanced Search  Database - CINAHL Complete | 30,057 |
| S62 | (MH "Cellular Phone") OR (MH "Smartphone") OR (MH "Mobile Applications") | Search modes - Boolean/Phrase | Interface - EBSCOhost Research Databases  Search Screen - Advanced Search  Database - CINAHL Complete | 6,408 |
| S63 | TI "mobile device*" OR AB "mobile device*" | Search modes - Boolean/Phrase | Interface - EBSCOhost Research Databases  Search Screen - Advanced Search  Database - CINAHL Complete | 1,159 |
| S64 | (MH "Virtual Reality") OR "Virtual reality" | Search modes - Boolean/Phrase | Interface - EBSCOhost Research Databases  Search Screen - Advanced Search  Database - CINAHL Complete | 4,960 |
| S65 | (MH "Online Systems") OR "online" | Search modes - Boolean/Phrase | Interface - EBSCOhost Research Databases  Search Screen - Advanced Search  Database - CINAHL Complete | 50,523 |
| S66 | (MH "Internet") OR "Internet" | Search modes - Boolean/Phrase | Interface - EBSCOhost Research Databases  Search Screen - Advanced Search  Database - CINAHL Complete | 54,274 |
| S67 | TI (web n1 based) OR AB (web n1 based) | Search modes - Boolean/Phrase | Interface - EBSCOhost Research Databases  Search Screen - Advanced Search  Database - CINAHL Complete | 11,621 |
| S68 | TI ( (world wide web or WWW or website*) ) OR AB ( (world wide web or WWW or website*) ) | Search modes - Boolean/Phrase | Interface - EBSCOhost Research Databases  Search Screen - Advanced Search  Database - CINAHL Complete | 13,458 |
| S69 | (MH "Electronic Mail") OR "e-mail" or "email" | Search modes - Boolean/Phrase | Interface - EBSCOhost Research Databases  Search Screen - Advanced Search  Database - CINAHL Complete | 10,044 |
| S70 | TI ( ((mobile or cellular or cell or smart) n1 (phone* or telephone*)) ) OR AB ( ((mobile or cellular or cell or smart) n1 (phone* or telephone*)) ) | Search modes - Boolean/Phrase | Interface - EBSCOhost Research Databases  Search Screen - Advanced Search  Database - CINAHL Complete | 4,268 |
| S71 | TI ( (e-health or ehealth or "electronic health") ) OR AB ( (e-health or ehealth or "electronic health") ) | Search modes - Boolean/Phrase | Interface - EBSCOhost Research Databases  Search Screen - Advanced Search  Database - CINAHL Complete | 9,913 |
| S72 | TI ( ("m-health" or mhealth or "mobile health") ) OR AB ( ("m-health" or mhealth or "mobile health") ) | Search modes - Boolean/Phrase | Interface - EBSCOhost Research Databases  Search Screen - Advanced Search  Database - CINAHL Complete | 1,665 |
| S73 | TI ( (("digital health" or digital) n3 intervention*) ) OR AU ( (("digital health" or digital) n3 intervention*) ) | Search modes - Boolean/Phrase | Interface - EBSCOhost Research Databases  Search Screen - Advanced Search  Database - CINAHL Complete | 91 |
| S74 | TI ( (interactive n1 ((health n1 communicat*) or video* or technolog* or multimedia)) ) OR AB ( (interactive n1 ((health n1 communicat*) or video* or technolog* or multimedia)) ) | Search modes - Boolean/Phrase | Interface - EBSCOhost Research Databases  Search Screen - Advanced Search  Database - CINAHL Complete | 862 |
| S75 | TI ( ((chat n1 room*) or chatroom*) ) OR AB ( ((chat n1 room*) or chatroom*) ) | Search modes - Boolean/Phrase | Interface - EBSCOhost Research Databases  Search Screen - Advanced Search  Database - CINAHL Complete | 237 |
| S76 | (MH "User-Computer Interface") | Search modes - Boolean/Phrase | Interface - EBSCOhost Research Databases  Search Screen - Advanced Search  Database - CINAHL Complete | 8,848 |
| S77 | TI "human computer interaction*" OR AB "human computer interaction*" | Search modes - Boolean/Phrase | Interface - EBSCOhost Research Databases  Search Screen - Advanced Search  Database - CINAHL Complete | 235 |
| S78 | TI (phone n1 app*) OR AB (phone n1 app*) | Search modes - Boolean/Phrase | Interface - EBSCOhost Research Databases  Search Screen - Advanced Search  Database - CINAHL Complete | 428 |
| S79 | S62 OR S63 OR S64 OR S65 OR S66 OR S67 OR S68 OR S69 OR S70 OR S71 OR S72 OR S73 OR S74 OR S75 OR S76 OR S77 OR S78 | Search modes - Boolean/Phrase | Interface - EBSCOhost Research Databases  Search Screen - Advanced Search  Database - CINAHL Complete | 143,724 |
| S80 | S21 OR S51 | Search modes - Boolean/Phrase | Interface - EBSCOhost Research Databases  Search Screen - Advanced Search  Database - CINAHL Complete | 462,839 |
| S81 | S61 AND S79 AND S80 | Limiters - English Language; Human  Search modes - Boolean/Phrase | Interface - EBSCOhost Research Databases  Search Screen - Advanced Search  Database - CINAHL Complete | 747 |

**CENTRAL**

## 1926 Trials matching on 'Smartphone* or “cell phone*” or “smart phone*” or “mobile device*” or “mobile app*” or “virtual reality” or online or internet or “web based” or “world wide web” or www or website* or email* or “e-mail*” or “electronic mail*” or “e-health” or ehealth or “electronic health” or “m-health” or mhealth or “mobile health” or ((digital health or digital) near/3 intervention*) or interactive or video* or technolog* or multimedia or “chat room*” or chatroom* or “phone app*” or “user computer interface” or “human computer interaction*” in Title Abstract Keyword AND Usability or ((intervention or app or e-mail or email or m-health or e-health or digital health or online or web*) near/5 (adopt* or uptake or retention or maintenance or participat* or engag* or usage attrition or usage or adher*)) or “process metric*” or “process evaluation*” or login* or (page* near/3 view*) or ((module* or session*) near/1 complet*) or (visit* near/3 website*) or (time near/3 online) or (user near/3 (engag* or experience* or enjoyment or attention or interest or affect or immersion or involvement)) in Title Abstract Keyword AND 'exercis* or gymnastics or “physical conditioning” or running or swimming or walking or “motor activity” or “physical education and training” or “physical fitness” or “cardiorespiratory fitness” or “life style” or lifestyle or “healthy ag?ing” or “healthy diet” or sport* or “physical activit*” or “physical inactivit*” or sedentary or “fitness class*” or “fitness regime*” or “fitness program*” or diet or nutrition* or “child nutrition science*” or fruit* or vegetable* or canteen* or “food service*” or calorie* or “energy intake” or “energy density” or “feeding behavio*” or “dietary intake*” or food or “carbonated beverage*” or “soft drink*” or soda or “sweetened drink” or “dietary fat*” or confectionar* or “school lunch*” or “school meal*” or “feeding program*” or cafeteria* in Title Abstract Keyword - (Word variations have been searched)'

**Academic Search Ultimate**

exercis* or gymnastics or “physical conditioning” or running or swimming or walking or “motor activity” or “physical education and training” or “physical fitness” or “cardiorespiratory fitness” or “life style” or lifestyle or “healthy ag?ing” or “healthy diet” or sport* or “physical activit*” or “physical inactivit*” or sedentary or “fitness class*” or “fitness regime*” or “fitness program*” or diet or nutrition* or “child nutrition science*” or fruit* or vegetable* or canteen* or “food service*” or calorie* or “energy intake” or “energy density” or “feeding behavio*” or “dietary intake*” or food or “carbonated beverage*” or “soft drink*” or soda or “sweetened drink” or “dietary fat*” or confectionar* or “school lunch*” or “school meal*” or “feeding program*” or cafeteria*

Usability or ((intervention or app or “e-mail” or email or “m-health” or “e-health” or “digital health” or online or web*) n5 (adopt* or uptake or retention or maintenance or participat* or engag* or usage or adher*)) or “process metric*” or “process evaluation*” or login* or (page* n3 view*) or ((module* or session*) n1 complet*) or (visit* n3 website*) or (time n3 online) or (user n3 (engag* or experience* or enjoyment or attention or interest or affect or immersion or involvement))

Smartphone* or “cell phone*” or “smart phone*” or “mobile device*” or “mobile app*” or “virtual reality” or online or internet or “web based” or “world wide web” or www or website* or email* or “e-mail*” or “electronic mail*” or “e-health” or ehealth or “electronic health” or “m-health” or mhealth or “mobile health” or ((digital health or digital) n3 intervention*) or interactive or video* or technolog* or multimedia or “chat room*” or chatroom* or “phone app*” or “user computer interface” or “human computer interaction*”

**Scopus**

## TITLE ( exercis*  OR  gymnastics  OR  "physical conditioning"  OR  running  OR  swimming  OR  walking  OR  "motor activity"  OR  "physical education and training"  OR  "physical fitness"  OR  "cardiorespiratory fitness"  OR  "life style"  OR  lifestyle  OR  "healthy ag?ing"  OR  "healthy diet"  OR  sport*  OR  "physical activit*"  OR  "physical inactivit*"  OR  sedentary  OR  "fitness class*"  OR  "fitness regime*"  OR  "fitness program*"  OR  diet  OR  nutrition*  OR  "child nutrition science*"  OR  fruit*  OR  vegetable*  OR  canteen*  OR  "food service*"  OR  calorie*  OR  "energy intake"  OR  "energy density"  OR  "feeding behavio*"  OR  "dietary intake*"  OR  food  OR  "carbonated beverage*"  OR  "soft drink*"  OR  soda  OR  "sweetened drink"  OR  "dietary fat*"  OR  confectionar*  OR  "school lunch*"  OR  "school meal*"  OR  "feeding program*"  OR  cafeteria* )  AND  ABS ( usability  OR  ( ( intervention  OR  app  OR  e-mail  OR  email  OR  m-health  OR  e-health  OR  digital  AND  health  OR  online  OR  web* )  AND  ( adopt*  OR  uptake  OR  retention  OR  maintenance  OR  participat*  OR  engag*  OR  usage  AND  attrition  OR  usage  OR  adher* ) )  OR  "process metric*"  OR  "process evaluation*"  OR  login*  OR  ( page*  AND  view* )  OR  ( ( module*  OR  session* )  AND  complet* )  OR  ( visit*  AND  website* )  OR  ( time  AND  online )  OR  ( user  AND  ( engag*  OR  experience*  OR  enjoyment  OR  attention  OR  interest  OR  affect  OR  immersion  OR  involvement ) ) )  AND  ABS ( smartphone*  OR  "cell phone*"  OR  "smart phone*"  OR  "mobile device*"  OR  "mobile app*"  OR  "virtual reality"  OR  online  OR  internet  OR  "web based"  OR  "world wide web"  OR  www  OR  website*  OR  email*  OR  "e-mail*"  OR  "electronic mail*"  OR  "e-health"  OR  ehealth  OR  "electronic health"  OR  "m-health"  OR  mhealth  OR  "mobile health"  OR  ( digital  AND  intervention* )  OR  ( ( interactive  OR  video*  OR  technolog*  OR  multimedia )  AND  health )  OR  "chat room*"  OR  chatroom*  OR  "phone app*"  OR  "user computer interface"  OR  "human computer interaction*" )  AND  ( LIMIT-TO ( LANGUAGE ,  "English " ) )  AND  ( LIMIT-TO ( EXACTKEYWORD ,  "Human " )  OR  LIMIT-TO ( EXACTKEYWORD ,  " Humans " ) )
