# Supplementary material for: Associations Between Digital Health Intervention Engagement, Physical Activity, and Sedentary Behavior: Systematic Review and Meta-analysis
Source: J Med Internet Res. 2021 Feb 19;23(2):e23180. doi: 10.2196/23180 (PMC8011420; doi:10.2196/23180)
Supplement: Multimedia Appendix 4 [file jmir_v23i2e23180_app4.docx]

**Multimedia Appendix 4**

Effect estimates were transformed to a standardized regression coefficient (B). The following transformation were made:

Log Odds —> d —> r

b ---> B

r = B

Where: d = standardised mean difference; r = correlation coefficient; b = Unstandardised regression coefficient; B = standardised coefficient.

The table below details the equations used for the transformations.


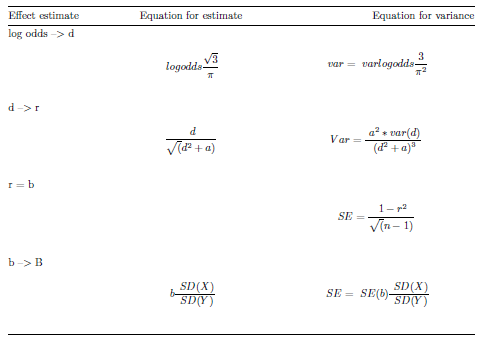


When SEs were missing they were calculated using the following formulas:


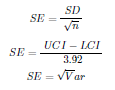


Where: SE = Standard error; d = Standardized mean difference; r = Correlation coefficient; b =

Standardized regression coefficient; B = Standardized regression coefficient; var = Variance; SD =

Standard deviation; UCI = Upper confidence interval; LCI = Lower confidence interval


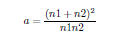


n1 = Sample size for group 1 n2 = Sample size for group 2

For studies where only a p-value was provided and no measure of variability, the p-value was used

to approximate the variance [1]. If an exact p-value was not provided (e.g. Ma and Lewis) and only

p<0.001 or p<0.01 was reported, a p-value of 0.0009 or 0.009, respectively, was assumed.

Three studies included in the meta-analysis assessed the relationship between physical activity and engagement by comparing the change in moderate to vigorous physical activity from baseline to follow-up. For these studies the difference was standardized using the baseline standard deviation rather than the standard deviation of change as recommended (1).

**References**

1. Morris SB, DeShon RP. Combining effect size estimates in meta-analysis with repeated measures and independent-groups designs. Psychological Methods. 2002;7(1):105-25.
