# Supplementary material for: Associations Between Digital Health Intervention Engagement, Physical Activity, and Sedentary Behavior: Systematic Review and Meta-analysis
Source: J Med Internet Res. 2021 Feb 19;23(2):e23180. doi: 10.2196/23180 (PMC8011420; doi:10.2196/23180)
Supplement: Multimedia Appendix 5 [file jmir_v23i2e23180_app5.docx]

**Multimedia Appendix 5**

**Table 1:** Characteristics of included studies

| **Author & study characteristics** | **Description of digital health intervention** | **Engagement Outcome(s)** | **Physical Activity or Sedentary Behaviour Outcome(s)** | **Association** | **Direction of significant association ^(e)^** |  |
| --- | --- | --- | --- | --- | --- | --- |
| **Author:** Bronner et al (2015) (1)  **Design^(a)^:** Cross-sectional  **n^(b)^=** 7  **Age^(c)^:** 29(9)  **Female:** 43%  **Recruitment method^(f)^**: non-ecological | **Type:** Exergame  **Description:**  Five different exergames across three different consoles (computer, Wii™ and Xbox 360®)  **Intervention target:** Experienced exergame players  **Length of exposure to DHI^(d)^:** 10-15hrs per game in one hour sessions. | Subjective experience  ‘engagement’ construct from The Exergame Questionnaire | Metabolic Equivalents via indirect calorimetry | Pearson’s product moment correlation coefficients.  r = 0.61 | + |  |
|  |  | Subjective experience ‘game-flow’ construct from The Exergame Questionnaire | Metabolic Equivalents via indirect calorimetry | Pearson’s product moment correlation coefficients.  r = 0.52 | + |  |
|  |  | Subjective experience ‘usability’ construct from The Exergame Questionnaire | Metabolic Equivalents via indirect calorimetry | Pearson’s product moment correlation coefficients.  *“There was no correlation between*  *usability scores and … METs”* | 0 |  |
| **Author:** Carr et al (2008) (2)  **Design^(a)^:** Cohort  **n^(b)^=** 14  **Age^(c)^:** 41.4(3.7)  **Female:** 79%  **Recruitment method^(f)^**: non-ecological | **Type:** Web-based  **Description:** Behaviour change program to increase physical activity. Includes modular style lessons, goal setting, performance feedback, a workbook, a study guide, interactive activities and read other participants behaviour change stories.  **Intervention target:** Inactive adults  **Length of exposure to DHI^(d)^:** 16 weeks | Activities completed | Steps (change in steps self-logged from a pedometer used for data collection only) | Multiple linear regression  “No other predictors, including measures of intervention compliance/engagement, entered into the regression model or  significantly explained additional variability for change in the amount of PA.” | 0 |  |
|  |  | Logins | Steps (change in steps self-logged from a pedometer used for data collection only) | Multiple linear regression  “No other predictors, including measures of intervention compliance/engagement, entered into the regression model or  significantly explained additional variability for change in the amount of PA.” | 0 |  |
| **Author:** Davies et al (2012) (3)  **Design^(a)^:** Cross-sectional  **n^(b)^=** 348  **Age^(c)^:**  18-34: 20%  35-44: 25%  45-54: 39.5%  >54: 15%  **Female:** 64%  **Recruitment method^(f)^**: Ecological | **Type:** Web-based  **Description:** Step count based intervention, with pedometer. Online step log feature. Interactive components, including I-Challenges which involves individuals choosing a selection of predefined monthly goals corresponding walking challenge.  **Intervention target:** Self-enrolled participants  **Length of exposure to DHI^(d)^:** 24 months | Activities completed (I-Challenges) | Steps (self-reported from pedometer which is a component of the intervention) dichotomized to greater or less than 10,000 steps. | Odds Ratio: 2.80 (95%CI: 1.45-5.40) | + |  |
|  |  | Activities completed (Team Challenges) | Steps (self-reported from pedometer which is a component of the intervention) dichotomized to greater or less than 10,000 steps. | Not reported in table, as it was not significant. | 0 |  |
|  |  | Activities completed (Virtual Walking Buddies) | Steps (self-reported from pedometer which is a component of the intervention) dichotomized to greater or less than 10,000 steps. | Not reported in table, as it was not significant. | 0 |  |
| **Author:** Edney et al 2018 (4, 5)  **Design^(a)^:** Cohort  **n^(b)^=** 89 (at baseline)  52  **Age^(c)^:** 35.1 (10.9)  **Female:** Unknown  **Recruitment method^(f)^**: Non-ecological | **Type:** Facebook (Group)  **Description:** A program to assist non-runners to undertake three-interval running sessions per week. Facebook group moderated with a daily message, with the aim of participants being able to run continuously for 30 minutes.  **Intervention target:** Australian Facebook users aged 18-50, not regular runners.  **Length of exposure to DHI^(d)^:** 8 weeks | Activities completed (sum of likes, comments, poll votes and photo uploads) | MVPA (change from baseline to 2 months in self-report from the Active Australia Survey) | Pearson correlation  r = -0.13 | 0 |  |
| **Author:** Edney et al 2019 (6)  **Design^(a)^:** Cohort  **n^(b)^=** 301  **Age^(c)^:** 42 (12)  **Female:** 74%  **Recruitment method^(f)^**: Non-ecological | **Type:** Smartphone App  **Description:** Two apps. Both encouraged users to take 10,000 steps per day for 100 days and sent daily reminders to self-monitor steps. ‘Gamified’ app included gamification, self-monitoring, Facebook-style newsfeed, challenges, leader board and unlockable gifts, e.g., send a high 5 to someone. ‘Basic App’ included only self-monitoring and a daily push notification to enter step counts.  **Intervention target:** Physically inactive Australian adults aged up to 65 years old using Facebook weekly.  **Length of exposure to DHI^(d)^:** 3 months | Activities Completed (total app use = each interaction with a feature of the app  (ie, step calendar, newsfeed, challenge page, gift page, and  friends page) was included as a single use.) | MVPA (change from baseline to 3 months in device-measured via GENEActiv) | Linear mixed models  “There was a weak, significant total app use-by-time interaction effect for objective PA (F_1,272=_4.5; P=.04)…, where higher total app use (individual-level engagement metric) was associated with greater increases in PA at 3-month follow-up.” | + |  |
|  |  | Activities Completed (total app use = each interaction with a feature of the app  (ie, step calendar, newsfeed, challenge page, gift page, and  friends page) was included as a single use.) | MVPA (change at 3 months in self-report via Active Australia Survey) | Linear mixed models  “There was a weak, significant total app use-by-time interaction effect for … self-reported PA (F_1,304_=6.56; P=.01), where higher total app use (individual-level engagement metric) was associated with greater increases in PA at 3-month follow-up.” | + |  |
| **Author:** Ferney et al 2009 (7)  **Design^(a)^:** Cohort  **n^(b)^=** 52  **Age^(c)^:** 52(4)  **Female:** 77%  **Recruitment method^(f)^**: Non-ecological | **Type:** Web-based  **Description:** Website providing 11 content areas, resources, links, goal setting tool, a database of local physical activity opportunities, calendar of events, map of walking trails, quiz, bulletin board and news section.  **Intervention target:** Adults aged 45-60, not meeting physical activity guidelines, and who reside in specific suburbs of Brisbane, Australia.  **Length of exposure to DHI^(d)^:** 26 weeks | Logins (dichotomised to users = two or more accesses of the website; and, non-user groups = less than two accesses of the website) | MVPA (Change in Self-report via the Active Australia Questionaire) | ANCOVA (3 time x 2 group)  p = 0.69 | 0 |  |
|  |  |  | Total Walking (Change in self-report via the Active Australia Questionaire) | ANCOVA (3 time x 2 group)  0.7 | 0 |  |
|  |  |  | Neighborhood walking (Change in self-report via the Active Australia Questionaire) | ANCOVA (3 time x 2 group)  0.09 | 0 |  |
|  |  |  | Community walking path (Change in self-report via the Active Australia Questionaire) | ANCOVA (3 time x 2 group)  0.05 | + |  |
| **Author:** Hansen et al (2012) (8)  **Design^(a)^:** Cross-sectional  **n^(b)^=** 3555 (baseline)  **Age^(c)^:** 51(14)  **Female:** 65%  **Recruitment method^(f)^**: Non-ecological | **Type:** Web-based  **Description:** A website with a personal profile, tailored advice, resources, discussion forum and monthly emails.  **Intervention target:** Physically inactive adults.  **Length of exposure to DHI^(d)^:** 6 months | Logins (trichotomized to no log on, log on once and log on more than once). | MVPA (self-report via IPAQ survey at 6 months) | Kruskal-Wallis test for differences between three groups.  p = <0.001 | + |  |
| **Author:** Hoj et al (2017) (9)  **Design^(a)^:** Cross-sectional  **n^(b)^=** 207  **Age^(c)^:**  18-25 (8.2%)  26-34 (45.9%)  35-54 (40.1%)  55-64 (5.3%)  65 or older (0.5%)  **Female:** 51%  **Recruitment method^(f)^**: ecological | **Type:** Smartphone Apps (any)  **Description:** Participants who had used a physical activity app in the last six months. Could be any physical activity apps.  **Intervention target:** USA residents who’ve used a physical activity app in the last 6 months.  **Length of exposure to DHI^(d)^:** various. | Subjective experience (Engagement and app likeability) | Composite Physical Activity Score (self-report) | Multiple regression  Coefficient (SE)= 0.40 (0.074) | + |  |
|  |  | Logins (App use Frequency of app use in the last 6 months) | Composite Physical Activity Score (self-report) | Multiple regression  Coefficient (SE)= -0.01 (0.067) | 0 |  |
| **Author:** Kwan et al (2013) (10)  **Design^(a)^:** Cohort  **n^(b)^=** 65  **Age^(c)^:** 18(1)  **Female:** 68%  **Recruitment method^(f)^**: Non-ecological | **Type:** Web-based  **Description:** Hosted within teaching website (Blackboard) with weekly topics on beliefs about physical activity, goal setting, action planning and behavioural maintenance.  **Intervention target:** Toronto University students living on campus  **Length of exposure to DHI^(d)^:** 6 weeks | Logins (low=1 or less; High= 2 or more) | MVPA (change in self-report via Behaviour Risk Factor Surveillance System) | ANOVA  (F_1_,_63_=1.54, P=.22, n_p_^2^=.03) | 0 |  |
|  |  |  |  |  |  |  |
| **Author:** Lewis et al (2008) (11)  **Design^(a)^:** Cross sectional and Cohort  **n^(b)^=** 147  **Age^(c)^:** Not reported  **Female:** 83%  **Recruitment method^(f)^**: non-ecological | **Type:** Web-based  **Description:** Two interventions, both websites, and associations are pooled over both. ‘Standard’ intervention included links to 6 publically available websites, monthly email prompts and asked to complete questionnaires. ‘Tailored’ intervention included monthly tailored feedback, tip of the day, goal setting, self-monitoring and educational materials.  **Intervention target:** Physically inactive adults.  **Length of exposure to DHI^(d)^:** 12 months | Activities completed (number of goals set) | MVPA (meeting PA guidelines at 12 months) | Odds Ratio  1.29 (95%CI: 1.14-1.47) | + |  |
|  |  | Logins | MVPA (change in self-report from baseline to 12 months via 7-Day Physical Activity recall) | Quintile Regression  t = 3.39 (P = <0.01) | + |  |
|  |  | Subjective Experience - Website usefulness rating | MVPA (change in self-report from baseline to 12 months via 7-Day Physical Activity recall) | Quintile Regression  t = 2.32 (P = <0.01) | + |  |
| **Author**  Lieber et al (2012) (12)  **Design^(a)^:** Cross-sectional  **n^(b)^=** 892  **Age^(c)^:**  18–24 (2.5%)  25–34 (14.0%)  35–44 (24.3%)  45–54 (34.1%)  55–64 (21.0%)  65–74 (3.7%)  >75 (0.5%)  **Female**100%**:**  **Recruitment method^(f)^**: Ecological | **Type:** Web-based  **Description:** Modular format, with weekly self-assessments, emails and progress logs.  **Intervention target:** Physically inactive females.  **Length of exposure to DHI^(d)^:** 12 weeks | Activities completed (weeks completed) | MVPA (self-report meeting guidelines via Colditz et al 1994 self-administered physical activity questionnaire) | Odds Ratio  1.05 (95% CI 1.01-1.09) | + |  |
| **Author:** Linke et al (2019) (13)  **Design^(a)^:** Cohort and cross sectional  **n^(b)^=** 104  **Age^(c)^:** 39 (11)  **Female:** 100%  **Recruitment method^(f)^**: Non-ecological | **Type:** Web-based  **Description:** Monthly online surveys, feedback, facts. And an online manual encouraging goal setting, self-monitoring, rewarding, problem-solving and social support.  **Intervention target:** Inactive Spanish speaking Latinas (females) aged up to 65 years old, with regular internet access, who had a functional level of hospitalized due to psychiatric disorder in the last 3 years.  **Length of exposure to DHI^(d)^:** 12 months | Time (time spent on website) | MVPA (via accelerometer) | Generalized linear mixed models  “Both log-ins and time spent on the website were significantly related to intervention success (achieving higher mean minutes of MVPA per week at 12-month follow-up, controlling for baseline:  b=.48, SE 0.20, P=.02 for objectively measured MVPA  and b=.74, SE 0.34, P=.03 for self-reported MVPA).” | + |  |
|  |  | Time (time spent on website) | MVPA (change in self-reported MVPA at 12 months via 7-day Physical Activity Recall survey) | See above. | + |  |
|  |  | Logins | MVPA (via accelerometer) | See above. | + |  |
|  |  | Logins | MVPA (change in self-reported MVPA at 12 months via 7-day Physical Activity Recall survey) | See above. | + |  |
|  |  | Time (time spent on website) | MVPA (meeting 150min week guideline at 12 months self-reported via 7-day physical activity recall) | Unknown statistical analysis method  “Furthermore, those meeting ACSM guidelines for PA at 12  months (≥150 minutes per week of self-reported MVPA) spent significantly more time on the website than those not meeting guidelines (35 vs 20 minutes over 12 months, P=.002).” | + |  |
|  |  | Activities completed (Goal setting, physical activity tips and physical activity reports) |  | Unknown statistical analysis method    “goal-setting features, personal PA reports, and PA tips were the most used  portions of the website. Higher use of these features was associated with greater success in the program (more minutes of self-reported MVPA at 12 months controlling for baseline).”  “Specifically, one additional use of these features per month over 12 months translated into an additional 34 minutes per week of  MVPA (goals feature), 12 minutes per week (physical activity tips), and 42 minutes per week (physical activity reports).” | + |  |
|  |  | Activities completed (Goal setting) | MVPA (change in self-reported MVPA at 12 months via 7-day Physical Activity Recall survey) | Generalized linear models  *b*=2.85  Standard Error: 1.38  P = 0.04 | + |  |
|  |  | Activities completed (Physical activity tips) | MVPA (change in self-reported MVPA at 12 months via 7-day Physical Activity Recall survey) | Generalized linear models  *b*=1.00  Standard Error: 0.82  P = 0.05 | + |  |
|  |  | Activities completed (Physical activity reports) | MVPA (change in self-reported MVPA at 12 months via 7-day Physical Activity Recall survey) | Generalized linear models  *b*=3.49  Standard Error: 1.28  P = 0.01 | + |  |
| **Author:** Ma et al (2018) (14)  **Design^(a)^:** Cohort  **n^(b)^=** 210  **Age^(c)^:** 26(9)  **Female:** 34%  **Recruitment method^(f)^**: Ecological | **Type:** Smartphone app (Pokémon Go)  **Description:** Existing users of Pokémon Go app, an virtual reality based app where participants travel to physical locations to interact with game features.  **Intervention target:** Pokémon Go users who play on an iPhone.  **Length of exposure to DHI^(d)^:** 21days following installation of the app. | Time (Number of days played) | Distance travelled (Change in daily walking and running distances from 14 days before game installed to 21 days after (km) (via iPhone Health App)) | Multi-level modelling  Beta = -0.005  P = <0.001 | - |  |
| **Author:** Maher et al **(**2015) (15)  **Design^(a)^:** Cohort  **n^(b)^=** 51 (baseline)  **Age^(c)^:**  18 to <25 (24%)  25 to <35 (33%)  35 to <45 (24%)  45 to 65 (20%)  **Female:** 73%  **Recruitment method^(f)^**: non-ecological | **Type:** Web-based (integrated with Facebook App)  **Description:** Team-based Facebook app to assist adults to increase physical activity. Encouraged to achieve 10,000 steps a day, working in teams of 3-8 exisiting Facebook friends, calender to log daily steps, dashboard for step logging progress, message board, team tally, daily tips to be active and weekly emails.  **Intervention target:** Inactive adults, existing Facebook users aged up to 65 years old.  **Length of exposure to DHI^(d)^:** 7 weeks | Logins (dichotomised to low = 18 or less logins; high = 19 or more logins) | MVPA (Change in Self-report via Active Australia Survey) | Generalised linear mixed models  (F_1,41_= 3.06, P =.04) | + |  |
| **Author:** Marcus et al (2007) (16)  **Design^(a)^:** Cohort  **n^(b)^=** 163 (at baseline)  **Age^(c)^:** 45(9)  **Female:** 83%  **Recruitment method^(f)^**: non-ecological | **Type:** Web-based  **Description:** Two interventions, associations use pooled data. ‘Standard’ included monthly questionnaires, self-monitoring, and links to external websites. Tailored arm includes education, motivational materials, goal setting, external links, email prompts bi-weekly on average, feedback, self-monitoring).  **Intervention target:** Inactive adults who didn’t drink more than 3 drinks per day for 5 or more days, were not pregnant, were not planning to move house, were not mentally ill in the last 6 months.  **Length of exposure to DHI^(d)^:** 12 months | Logins (log-transformed) | MVPA (median minutes change in Self-report via 7-day physical activity recall) | Quantile Regression  B = 34.32 (95%CI: 14.33-54.31) | + |  |
| **Author:** Marquet et al (2018) (17)  **Design^(a)^:** Cross-sectional  **n^(b)^=** 47  **Age^(c)^:** 19.6 (SD not provided)  **Female:** 50%  **Recruitment method^(f)^**: Non-ecological and ecological. | **Type:** Smartphone App (Pokémon Go)  **Description:** Both existing users and new users of Pokémon Go app, a virtual reality based app where participants travel to physical locations to interact with game features.  **Intervention target:** North Carolina University undergraduate students  **Length of exposure to DHI^(d)^:** 1 week | Time (spent playing Pokémon Go per day) | Steps (recorded via phone using external app ‘PACO’. These are then self-logged daily using ecological momentary surveys) | Correlations  “When focused only on those who self-identified as Pok_emon GO players (n = 47), however, a relationship was found using an ANCOVA, between the actual playing minutes per day and the number of steps. The following partial correlation was found r = 0.176, p < .05, when we controlled for gender, OS of the device, ethnicity, and self-reported energy expenditure.” | + |  |
|  |  | Number of active playing episodes accumulated (n=1) | Steps (recorded via phone using external app ‘PACO’. These are then self-logged daily using ecological momentary surveys) | Linear mixed-effects regression  Mean difference (steps)= 206  Std. Error = 353  df = 73  p = 0.56 | 0 |  |
|  |  | Number of active playing episodes accumulated (n=2) | Steps (recorded via phone using external app ‘PACO’. These are then self-logged daily using ecological momentary surveys) | Linear mixed-effects regression  Mean difference (steps)= 224  Std. Error = 460  df = 183  p = 0.63 | 0 |  |
|  |  | Number of active playing episodes accumulated (n=3) | Steps (recorded via phone using external app ‘PACO’. These are then self-logged daily using ecological momentary surveys) | Linear mixed-effects regression  Mean difference (steps)= 1527  Std. Error = 599  df = 63  p = 0.013 | + |  |
|  |  | Number of playing episodes accumulated (n=1) | Steps (recorded via phone using external app ‘PACO’. These are then self-logged daily using ecological momentary surveys) | Linear mixed-effects regression  Mean difference (steps)= 75  Std. Error = 335  df =79  p = 0.83 | 0 |  |
|  |  | Number of playing episodes accumulated (n=2) | Steps (recorded via phone using external app ‘PACO’. These are then self-logged daily using ecological momentary surveys) | Linear mixed-effects regression  Mean difference (steps)= -253  Std. Error = 453  df = 216  p = 0.58 | 0 |  |
|  |  | Number of playing episodes accumulated (n=3) | Steps (recorded via phone using external app ‘PACO’. These are then self-logged daily using ecological momentary surveys) | Linear mixed-effects regression  Mean difference (steps)=875  Std. Error = 463  df = 107  p = 0.06 | 0 |  |
| **Author:** Rebar et al (2016) (18)  **Design^(a)^:** Cohort  **n^(b)^=** 514 (intention to treat)  **Age^(c)^:** 46 (15)  **Female:** 69%  **Recruitment method^(f)^**: Non-ecological | **Type:** Web-based  **Description:** Two interventions (pooled for analyses of associations). ‘MyPAA’ personalised website, tailored content, text, video and graphs, action plans. ‘Walk 2.0’ interactive website, feedback, social media integration, resources, self-monitoring steps.  **Intervention target:** Adults clicking on an advert on Google or Facebook ‘Feeling Down? Get Active! Free access to web-based physical activity program for participating in research study’  **Length of exposure to DHI^(d)^:** 3 months | Time (dichotomized to 4 minutes or less) | MVPA (change in physical activity from baseline to 1 months self-reported via Active Australia Survey) | Linear Mixed Models  Y=2.33 (95%CI: 0.09 to 4.64)  P<0.05 | + |  |
|  |  | Time (dichotomized to 4 minutes or less) | MVPA (change in physical activity from baseline to 3 months self-reported via Active Australia Survey) | Linear Mixed Models  Y=0.51 (95%CI: -1.77 to 2.72)  P>0.05 | 0 |  |
|  |  | Logins (dichotomized to low = 1 or less; high= 2 or more) | MVPA (change in physical activity from baseline to 1 months self-reported via Active Australia Survey) | Linear Mixed Models  Y=3.18 (95%CI: 1.15 to 5.07)  P<0.05 | + |  |
|  |  | Logins (dichotomized to low = 1 or less; high= 2 or more) | MVPA (change in physical activity from baseline to 3 months self-reported via Active Australia Survey) | Linear Mixed Models  Y=2.04 (95%CI: 0.29 to 3.84)  P<0.05 | + |  |
| **Author:** Wanner et al (2009) (19)    **Design^(a)^:** Cohort  **n^(b)^=** At baseline.  IG: 681  SU:162  **Age^(c)^:**  IG: 44 (13)  SU: 39 (13)  **Female:**  IG: 75%  SU:71%  **Recruitment method^(f)^**: Non-ecological and ecological. | **Type:** Web-based  **Description:** Freely available website available in German, French and Italian. Choice of modules to increase physical activity through every day activities, endurance training, strength and flexibility. Feedback via questionnaires. Fact sheets. Email reminders at 9,10, 11 months.  **Intervention target:** Adults aged 30-60. Advertising in newspapers, magazines and on the internet. Media-recruited participants were allocated to IG (Intervention Group). Spontaneous users (SU) were those who visited the website directly, and were recruited from the website, but may have seen an advertisement for the study to visit the website.  **Length of exposure to DHI^(d)^:** 13 months | Time (minutes spent in the tailored intervention) | MVPA (change in physical activity from baseline to 13 months self-reported via physical activity 4-item questionnaire)  (unadjusted model) | Linear Regression  Coefficient = 1.13 (95%CI: 0.09 to 2.17)  P = .03 | + |  |
|  |  | Time (minutes spent in the tailored intervention) | MVPA (change in physical activity from baseline to 13 months self-reported via physical activity 4-item questionnaire)  (adjusted for age, gender and BMI category) | Linear Regression  Coefficient = 1.07 (95%CI: 0.004 to 2.13)  P = .049 | + |  |
|  |  | Time (minutes spent in the tailored intervention) | MVPA (change in physical activity from baseline to 13 months self-reported via physical activity 4-item questionnaire) (adjusted for age, gender, BMI category and stage of change) | Linear Regression  Coefficient = 0.58 (95%CI: -0.43 to 1.59)  P = .26 | 0 |  |
| **Author:** Xian et al (2017) (20)  **Design^(a)^:** Cohort  **n^(b)^=** 167 (at baseline)  **Age^(c)^:** Median (IQR): 25 (21 to 29)  **Female:** 48%  **Recruitment method^(f)^**: Ecological | **Type:** Smartphone App (Pokémon Go)  **Description:** Existing users of Pokémon Go app, a virtual reality based app where participants travel to physical locations to interact with game features.  **Intervention target:** Existing users of Pokémon Go**,** using an iPhone, across multiple continents.  **Length of exposure to DHI^(d)^:** 3 weeks | Activities completed (XP points earned in app) | Steps (extracted directly from the iPhone Health App) | Ordinal least squares regression  “On average, every 10 000 XP points gained in Pokémon Go were associated with 2134 additional steps per day (95% CI, 1673–2595; P<0.001 [R2=0.33])” | + |  |
| **Table footnotes:**  (a) Studies were considered cohort if either the engagement measures, or physical activity or sedentary behaviour measures, used with the associations were change over time.  (b) The sample size presented in the table is that at the association unless otherwise noted.  (c) Age is presented as the mean (SD) of the total study sample at baseline unless otherwise noted.  (d) This is total length of DHI irrespective of when the association measure was taken;  (e) Each study was summarized as either ‘+’, ‘-‘ or ‘0’. ‘+’ were assigned to studies where the point estimate and confidence interval supported the hypothesis that higher engagement is associated with higher physical activity or reduced sedentary behaviour. ‘0’ were assigned to studies where the point estimate and confidence interval had inconclusive findings. ‘-‘ were assigned to studies where the association point estimate and confidence interval rejected the hypothesis. We assigned either ‘+’ or ‘-‘ to studies without point estimates or confidence intervals which reported “significant” association findings. We assigned ‘0’ to studies without point estimates or confidence intervals which reported “non-significant findings”.  (f) Studies were either ecological or non-ecological. Ecological studies were those that recruited ‘real-world’ participants where there are no repeated contacts with research staff, comprehensive assessments or incentives.  Abbreviations: DHI: Digital Health Intervention; MVPA: Moderate to vigorous physical activity. | | | | | | |

1. Bronner S, Pinsker R, Adam Noah J. Physiological and psychophysiological responses in experienced players while playing different dance exer-games. Computers in Human Behavior. 2015;51:34-41.

2. Carr LJ, Bartee RT, Dorozynski C, Broomfield JF, Smith ML, Smith DT. Internet-delivered behavior change program increases physical activity and improves cardiometabolic disease risk factors in sedentary adults: Results of a randomized controlled trial. Preventive medicine. 2008;46(5):431-8.

3. Davies C, Corry K, Van Itallie A, Vandelanotte C, Caperchione C, Mummery WK. Prospective Associations Between Intervention Components and Website Engagement in a Publicly Available Physical Activity Website: The Case of 10,000 Steps Australia. J Med Internet Res. 2012;14(1):e4.

4. Edney S, Looyestyn J, Ryan J, Kernot J, Maher C. Posts, pics, or polls? Which post type generates the greatest engagement in a Facebook physical activity intervention? Translational behavioral medicine. 2018;8(6):953-7.

5. Looyestyn J, Kernot J, Boshoff K, Maher C. A Web-Based, Social Networking Beginners’ Running Intervention for Adults Aged 18 to 50 Years Delivered via a Facebook Group: Randomized Controlled Trial. J Med Internet Res. 2018;20(2):e67.

6. Edney S, Ryan JC, Olds T, Monroe C, Fraysse F, Vandelanotte C, et al. User Engagement and Attrition in an App-Based Physical Activity Intervention: Secondary Analysis of a Randomized Controlled Trial. J Med Internet Res. 2019;21(11):e14645.

7. Ferney SL, Marshall AL, Eakin EG, Owen N. Randomized trial of a neighborhood environment-focused physical activity website intervention. Preventive medicine. 2009;48(2):144-50.

8. Hansen AW, Grønbæk M, Helge JW, Severin M, Curtis T, Tolstrup JS. Effect of a Web-Based Intervention to Promote Physical Activity and Improve Health Among Physically Inactive Adults: A Population-Based Randomized Controlled Trial. J Med Internet Res. 2012;14(5):e145.

9. Hoj TH, Covey EL, Jones AC, Haines AC, Hall PC, Crookston BT, et al. How Do Apps Work? An Analysis of Physical Activity App Users’ Perceptions of Behavior Change Mechanisms. JMIR Mhealth Uhealth. 2017;5(8):e114.

10. Kwan M, Faulkner G, Bray S. Evaluation of Active Transition, a Website-Delivered Physical Activity Intervention for University Students: Pilot Study. JMIR Res Protoc. 2013;2(1):e16.

11. Lewis B, Williams D, Dunsiger S, Sciamanna C, Whiteley J, Napolitano M, et al. User attitudes towards physical activity websites in a randomized controlled trial. Preventive medicine. 2008;47(5):508-13.

12. Lieber SB, Redberg RF, Blumenthal RS, Gandhi A, Robb KJ, Mora S. A National Interactive Web-Based Physical Activity Intervention in Women, Evaluation of the American Heart Association Choose to Move Program 2006&#x2013;2007. American Journal of Cardiology. 2012;109(12):1754-60.

13. Linke SE, Dunsiger SI, Gans KM, Hartman SJ, Pekmezi D, Larsen BA, et al. Association Between Physical Activity Intervention Website Use and Physical Activity Levels Among Spanish-Speaking Latinas: Randomized Controlled Trial. J Med Internet Res. 2019;21(7):e13063.

14. Ma BD, Ng SL, Schwanen T, Zacharias J, Zhou M, Kawachi I, et al. Pokémon GO and Physical Activity in Asia: Multilevel Study. J Med Internet Res. 2018;20(6):e217.

15. Maher C, Ferguson M, Vandelanotte C, Plotnikoff R, De Bourdeaudhuij I, Thomas S, et al. A Web-Based, Social Networking Physical Activity Intervention for Insufficiently Active Adults Delivered via Facebook App: Randomized Controlled Trial. J Med Internet Res. 2015;17(7):e174.

16. Marcus BH, Lewis BA, Williams DM, Dunsiger S, Jakicic JM, Whiteley JA, et al. A comparison of Internet and print-based physical activity interventions. Archives of internal medicine. 2007;167(9):944-9.

17. Marquet O, Alberico C, Hipp AJ. Pokémon GO and physical activity among college students. A study using Ecological Momentary Assessment. Computers in Human Behavior. 2018;81:215-22.

18. Rebar AL, Boles C, W. Burton N, Duncan MJ, Short CE, Happell B, et al. Healthy mind, healthy body: A randomized trial testing the efficacy of a computer-tailored vs. interactive web-based intervention for increasing physical activity and reducing depressive symptoms. Mental Health and Physical Activity. 2016;11:29-37.

19. Wanner M, Martin-Diener E, Braun-Fahrländer C, Bauer G, Martin BW. Effectiveness of Active-Online, an Individually Tailored Physical Activity Intervention, in a Real-Life Setting: Randomized Controlled Trial. J Med Internet Res. 2009;11(3):e23.

20. Xian Y, Xu H, Xu H, Liang L, Hernandez Adrian F, Wang Tracy Y, et al. An Initial Evaluation of the Impact of Pokémon GO on Physical Activity. Journal of the American Heart Association. 2017;6(5):e005341.
